# Exploring 2‐D σ−σ* Conjugation in Cyclic Polysiloxane Copolymers

**DOI:** 10.1002/marc.70348

**Published:** 2026-06-22

**Authors:** Zijing Zhang, Cecilia Pilon, Pimjai Pimbaotham, Siriporn Jungsuttiwong, Aleksander Rebane, Richard M. Laine

**Affiliations:** ^1^ Dept. of Materials Science and Engineering University of Michigan Ann Arbor Michigan USA; ^2^ Dept. of Chemistry University of Michigan Ann Arbor Michigan USA; ^3^ Department of Chemistry and Center of Excellence for Innovation in Chemistry Faculty of Science Ubon Ratchathani University Ubon Ratchathani Thailand; ^4^ Montana State University Bozeman Montana USA; ^5^ Macromolecular Science and Engineering Center University of Michigan Ann Arbor Michigan USA

**Keywords:** conjugated system, copolymer, electron transfer, excited state, heck reaction, photochemistry, polymer chemistry, polymer, substituent, thiophene

## Abstract

Here, we aim to clearly define phenomena previously termed unconventional conjugation involving conjugation across Si─O─Si bonds. The goal is to define operative mechanisms and provide general routes to easily accessible Si─O─Si containing copolymers using commercially available [vinylMeSiO]_4_, Vy_4_D_4_ (Vy = vinyl consistent with previous reports). Thus, 1:1 Vy_4_D_4_:Ar copolymers synthesized using Heck catalytic cross coupling (DPs 5–20) exhibit absorption λ_max_ typical of aromatic groups with weak, red‐shifted shoulders now recognized as σ‐σ* transitions. Emission λ_max_ redshift 40–80 nm from model compounds. Substituent dependent Ar’ substitution at remaining ring vinyls modulates emission photophysics. Thiophene co‐monomers in the main chain or as “decorated” substituents present the most red‐shifted, lowest‐energy excited states. In undecorated main chain copolymers, σ‐σ* conjugation appears operative. In decorated systems, three mechanisms are possible. Conjugation via the ring to the main chain, possible Förster‐type energy transfer involving decorated substituents or both. Excited state lifetime emission studies reveal two different emitting states, suggesting both mechanisms operate. Further proof is obtained on mixing copolymers with F_4_TCNQ. As with traditional conjugated polymers, formation of F_4_TCNQ^−.^ radical anion indicates electron transfer only seen when conjugation exists. In the current studies, F_4_TCNQ^−.^ forms for both undecorated and decorated copolymers indicating conjugation and σ‐σ* transitions. Most important, decorated Vy_4_D_4_ or [Vy_4_D_4_]Ar’_2‐5_ systems *not part of a copolymer* also form the radical anion F_4_TCNQ^−.^ implying that even simple, substituted cyclomers can exhibit σ‐σ* conjugation, suggesting multiple families of easily accessible σ‐σ* conjugated systems. Further proof comes from theoretical modeling, indicating the cyclomer flattens significantly in the excited state. Thus, decorated {[Vy_4_D_4_]Ar’_2‐4_}‐Ar‐ copolymers offer what can be termed 2‐D conjugation. One question remains: Do the conjugated cyclomers exhibit “ring current” in the excited state via σ‐σ* conjugation?

## Graphical abstract

We find σ−σ * conjugation in copolymers synthesized by reacting the cyclomer [vinylMeSiO]_4_ in a 1:1 ratio with various Br‐Ar‐Br. Further, on modifying the remaining unsubstituted viny groups with Br‐Ar’, we change the main chain photophysics. However, as a test, we also appended Br‐Ar’ groups on the cylomer alone observing σ−σ * conjugation coincident with significant flattening of the ring revealed as 140° bond angles perhaps suggesting ring current.

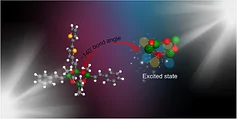

## 1 Introduction

**FIGURE 1:**
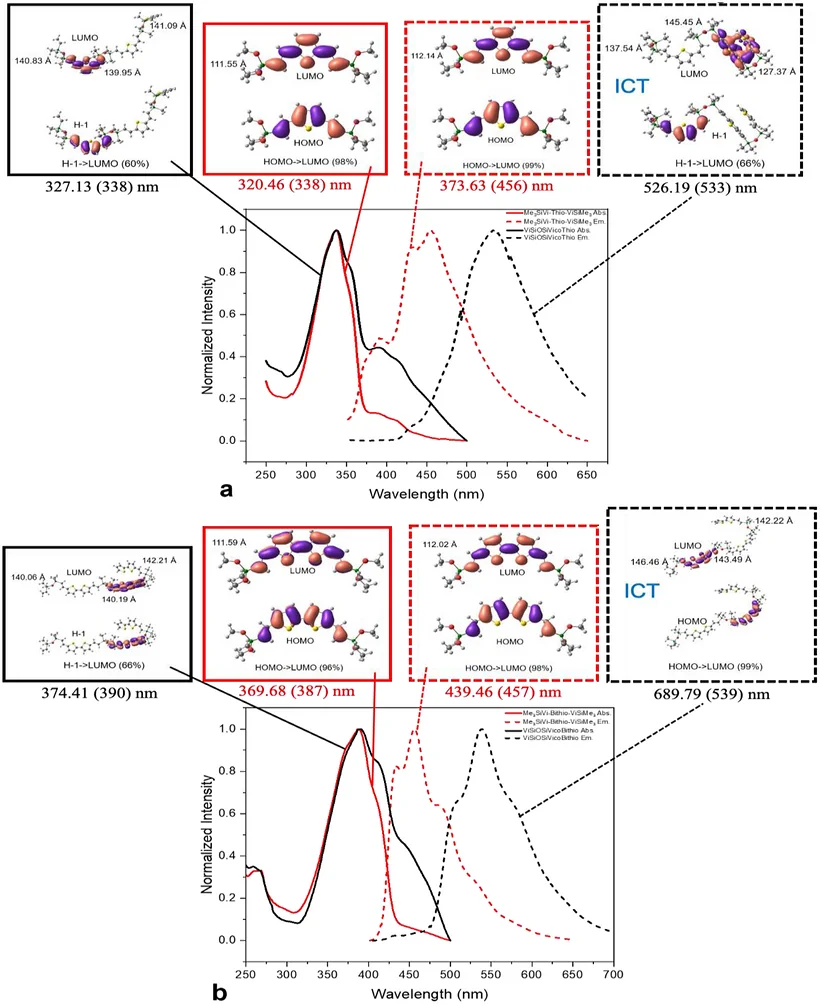
Normalized absorption (solid lines) and emission (dashed lines) and corresponding silane model compounds found and calculated spectra and structural conformations for (a) VySiOSiVy‐coThio (black) and (MeO)_2_SiMeVy‐Thio‐VySiMe(OMe)_2_ (red). (b) for VySiOSiVycoBithio (black) and model compound (MeO)_2_SiMeVy‐Bithio‐VySiMe(OMe)_2_ (red) [3].

We recently reported identifying a novel form of conjugation in polysiloxane copolymers wherein conjugation involves excitation from a σ bond ground state to a σ* excited state coincident with changes in Si─O─Si bond angles from 110°–112° in model compounds to 130°–140° in the copolymer ground state and to up to 150° in the excited state, as illustrated in Figure 1 [1, 2, 3].

**SCHEME 1:**
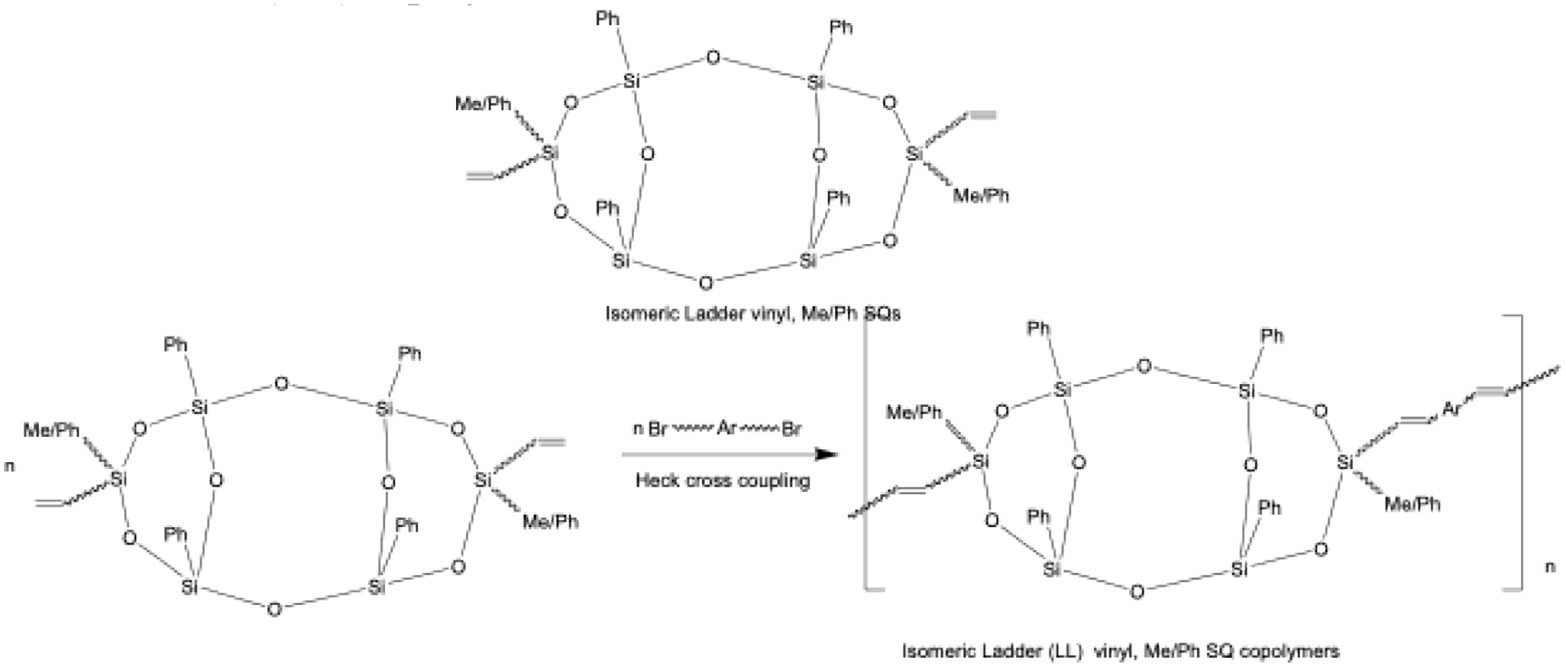
vinyl‐LL‐vinyl [6] isomers provide isomeric mixtures of derived LL copolymers [1].

**FIGURE 2:**
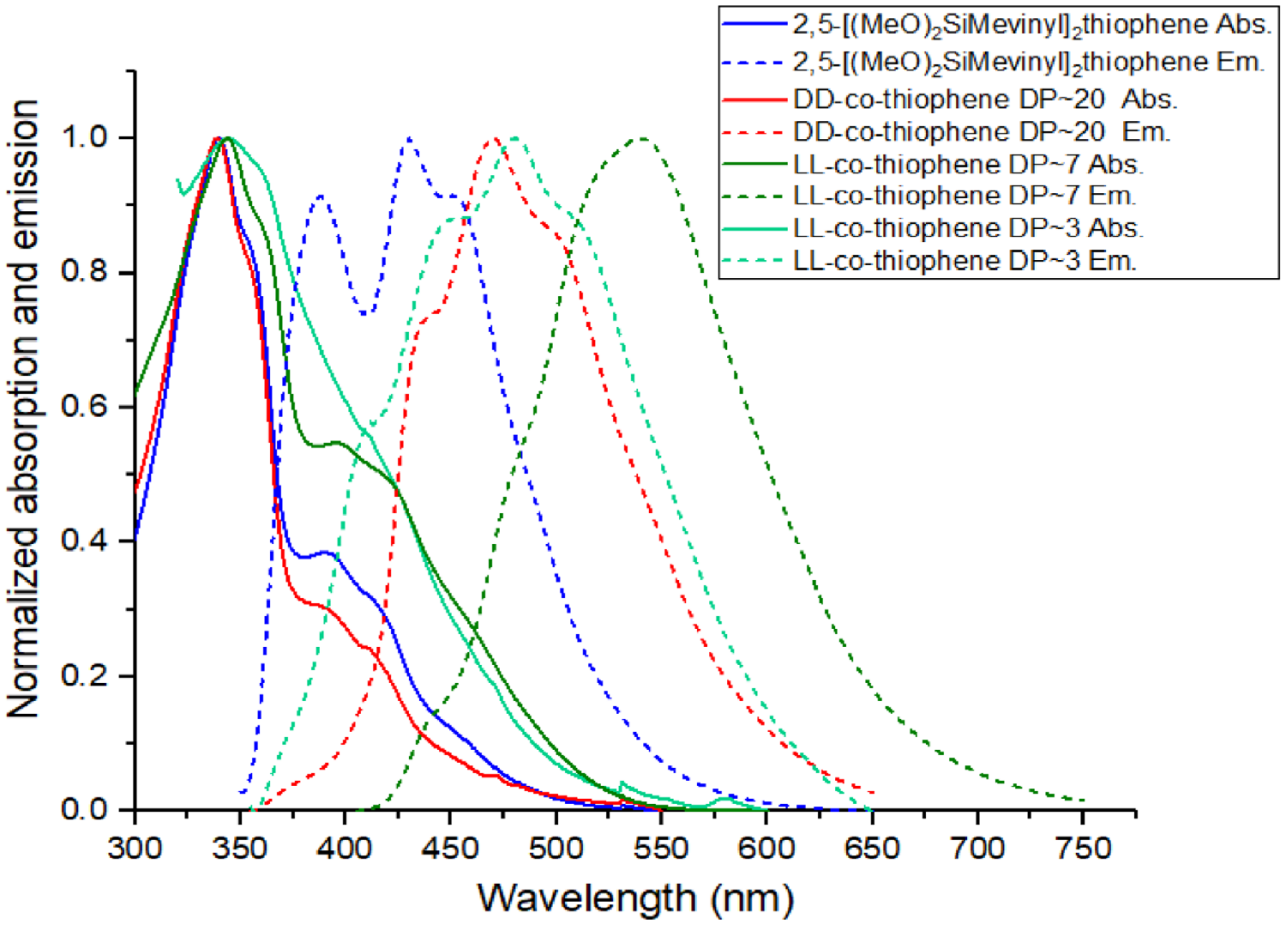
Steady‐state UV–vis spectra of 2,5‐[(MeO)_2_Sivinyl]_2_thiophene, DD‐co‐thiophene, short (DP∼3) and long (DP∼7) LL‐co‐thiophene in CH_2_Cl_2_ [4].

This surprising finding actually provides an explanation for previously published photophysics on ladder (LL) copolymers per Scheme 1 and Figure 2, wherein, despite having no silsesquioxane cage, the ladder copolymers offer red‐shifted λ_max_ actually shifted further red than the analogous double decker (DD) copolymers [4] at shorter DPs!

We previously labeled such behavior “unconventional conjugation” writing an invited review on the subject, but without offering a clear cut explanation for the observed photophysics [6]. We now recognize that absorption shoulders exhibited at 370–440 nm (Figure 2) are as expected for σ−σ* absorptions given the weak oscillator strength anticipated for σ−σ* transitions. *A further realization is that the ladder copolymers seem also to introduce a second dimension (2‐D) to* σ−σ* conjugation given that they are relatively rigid planar structures.

**SCHEME 2:**
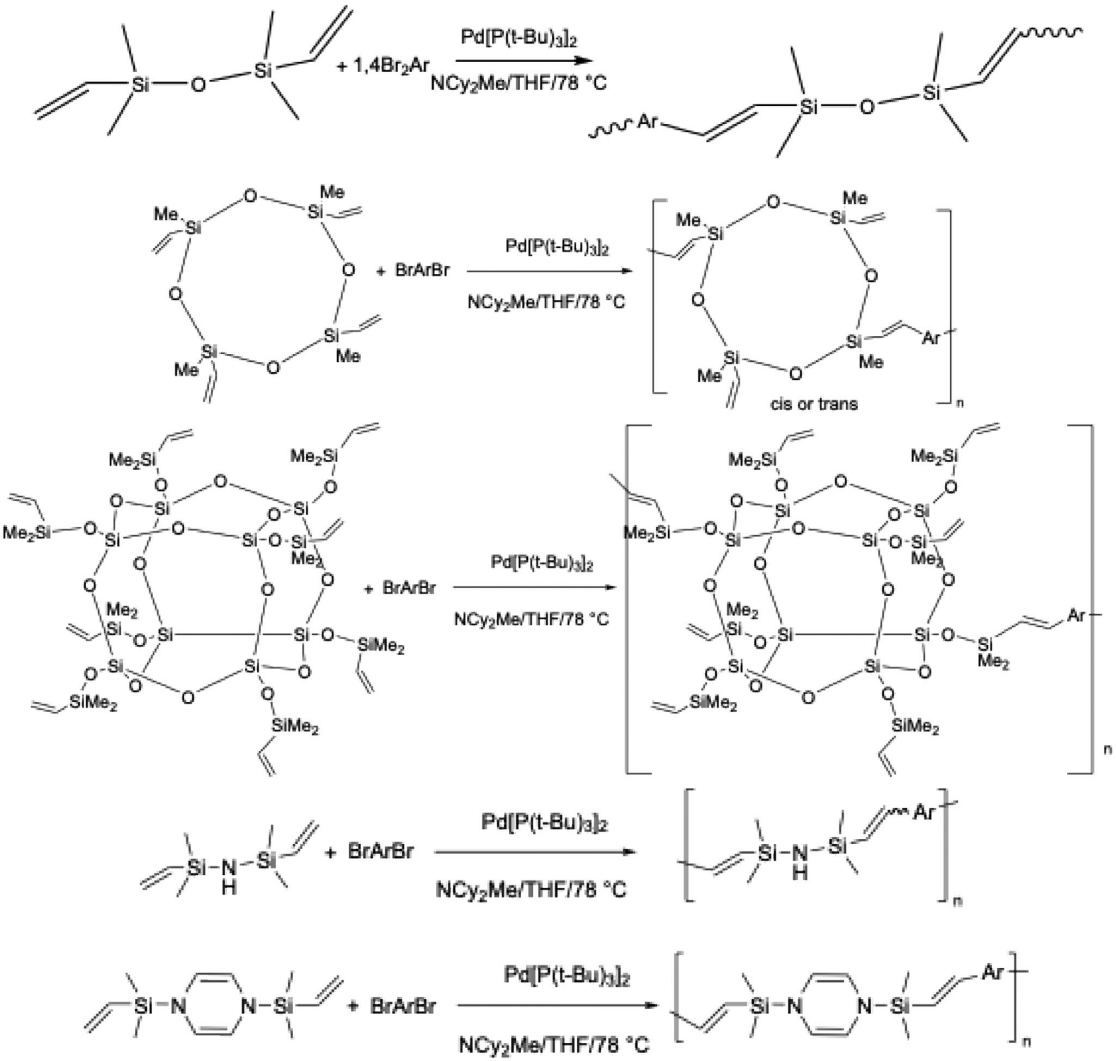
Potential families of copolymers that may offer σ−σ* conjugation.

After some reflection, we can now suggest new families of copolymers anticipated to offer 2‐D σ−σ* conjugation that greatly expand the utility of this unexpected conjugation mechanism, Scheme 2, if proven correct.

Of the possible sets of copolymers, the easiest to explore would be based on commercially available [MeVinylSiO]_4_. Thus, 1:1 Heck catalytic cross coupling with Br‐Ar‐Br copolymers should provide “beads on a chain” copolymers [7, 8, 9] (likely branched) where two vinyl groups remain that can be further modified to adjust photophysical properties, introducing “2‐D” conjugation. These systems also offer potential access to flexible thin films on crosslinking the remaining vinyls while retaining their photophysical properties, perhaps for display or hybrid photovoltaic applications [10].

The Q_8_ cage system would represent an effort to explore σ−σ* conjugation in a 3‐D system, but also assess the potential for conjugation across several linked Si─O─Si units in a rigid cage structure somewhat reminiscent of the rigid LL systems[5]. The silazane systems would allow extension to nitrogen analogs.

**SCHEME 3:**
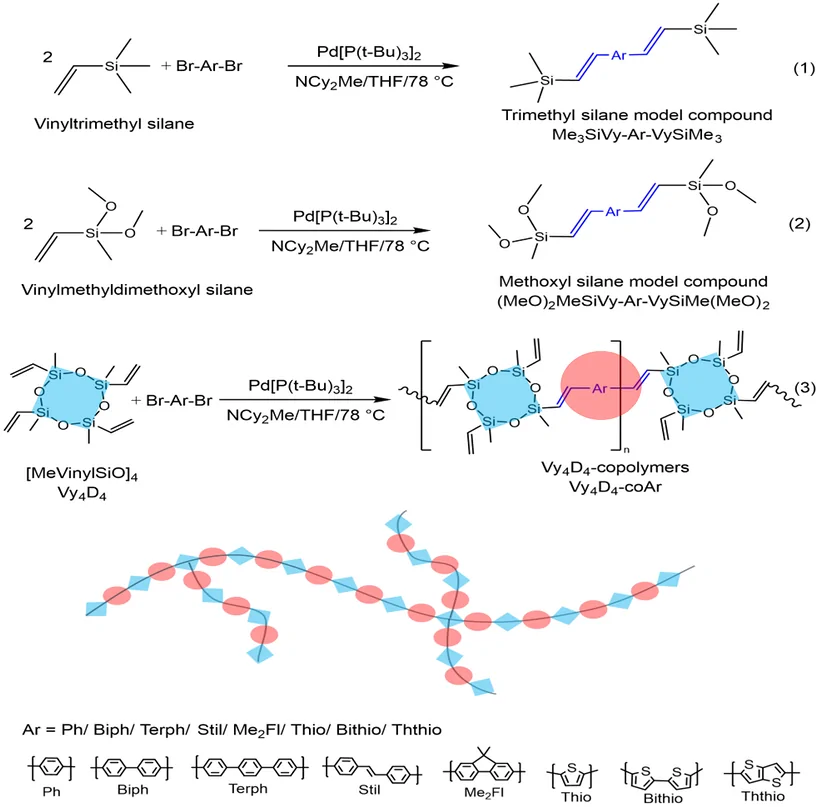
Syntheses of (1) trimethyl silane model compounds, (2) methoxy silane model compounds, and (3) branched Vy_4_D_4_‐copolymers.

These concepts provide the motivation for the work reported here, which begins with the synthesis of 1:1 [MeVinylSiO]_4_:‐Ar‐ copolymers (see Supporting Information for experimental and synthesis data, Table S1). Their photophysical properties are presented in Table S2 and compared to model compounds [(MeO)_2_MeSiVinyl‐Ar‐VinylSiMe(MeO)_2_] synthesized previously with the addition of Me_3_SiVinyl‐Ar‐VinylSiMe_3_ as the simplest model with only π‐π* transitions accessible, Scheme 3.

As illustrated in Scheme 3, the resulting 1:1 copolymers are expected to consist of beads on a chain structures with branching. As discussed below, these compounds offer red‐shifted emission λ_max_ relative to model compounds, indicating σ‐σ* conjugation across segments of the siloxane ring consistent with the linear siloxane copolymer photophysics [11]. *Most important to the present study is the opportunity to modify the initially found photophysics by appending groups to the approximately two vinyl groups per ring that remain, introducing “2‐D” conjugation*.

One might anticipate modifying the backbone double bonds via catalytic crosscoupling [11]; however, the copolymer vinyl groups are difficult to access both because of double substitution and polymer coiling, creating sufficient steric inhibition to greatly limit their reactivity. However, the remaining ring vinyl groups are accessible.

Thus, in a second, post‐functionalzation step, we introduce pendant conjugated groups (via Ar’‐Br) at positions remote from the continuous copolymer chains to probe their influence on main‐chain conjugation. Although these moieties were separated by “insulating” Si─O─Si ring units, the resulting “decorated” copolymers exhibit modified photophysics, implying conjugation across and around the ring. However, a much simpler explanation exists that the new photophysics arises based on Förster π−π* energy transfer (FRET) as assessed below [12, 13].

## 2 Results and Discussion

The objectives of the present work are to explore introducing ring structures into siloxane copolymers to add functionality while also adding a second dimension to σ−σ* conjugation.

### 2.1 Syntheses of Model Compounds and Vy_4_D_4_‐Copolymers

Reaction (1) and previously published reactions [1, 2] in Scheme 3 illustrate general efforts to synthesize models with the simplest π−π^*^ conjugated structures for comparison with copolymers synthesized via reaction (3). All reactions employed Heck catalytic cross‐coupling, as detailed in the experimental section in the Supporting Information.

All disubstituted model silanes were characterized by GPC (Table S3 and Figures S1–S9), FTIR (Table S4, Figures S34, and S35), and NMR (Table S5). Dispersities (Đ) for all silane model compounds are ∼1, indicating the formation of monomers. Table S4 summarizes representative FTIR spectra. Model silanes present peaks for νC─H ≈ 2950 cm^−1^ and νC═C ≈1500 cm^−1^ arising from the addition of aromatic moieties to vinylSiMe_3_, with the strongest peaks for νSi─C ≈ 1250 and 780 to 870 cm^−1^.

Vy_4_D_4_ derived copolymers were characterized in detail using GPC, TGA, FTIR, and NMR as presented in the Supporting Information. Besides peaks for νC─H, νC═C, and νSi─C described above, FTIR spectra (Table S4, Figure S36, and S37) present peaks for ν Si─O ≈1070 cm^−1^, indicating successful copolymerization with the introduction of the siloxane ring backbone. Figures S10–S17 display GPC traces for purified Vy_4_D_4_‐copolymers. GPC analysis (Table S1) indicates formation of oligomers with DPs ranging from 24 to 146 co‐monomer units. The observed dispersities (Đ ≥ 2) are consistent with step‐growth polymerization. ^1^H NMR of all copolymers displays chemical shifts for methyl groups ≈ 0.4 ppm, unreacted vinyl groups appear at 5.8–6.1 ppm with substituted vinyl groups at 6.1–7.1 ppm, thiophene groups ≈7.1 ppm, and phenyl groups around 7.4 ppm: Table S6, Figures S43–S50, Table S7, and Figures S51–S57 record ^13^C NMRs for vinyl, phenyl, and thiophene groups.

TGA results are recorded in Figures S58–S65 and Table S1. Decomposition temperatures (T_d5%_) in air for the copolymers are ≈ 400°C, suggesting high thermal stability. Notably, the experimentally determined ceramic yields (CYs) are lower than the theoretical values. This discrepancy may be attributed to the well‐known backbiting degradation mechanism of polysiloxane polymers[14]. Another reason could be multiple attachment of aromatic moieties to the four available vinyl groups/ring, resulting in partially branched oligomers and thus lower CYs.

### 2.2 Photophysical Properties of Model Compounds and Vy_4_D_4_‐Copolymers

Table S2 summarizes the steady‐state absorption and photoluminescence characteristics of synthesized Vy_4_D_4_‐copolymers and corresponding model compounds. Emission spectra were measured using excitation wavelengths matched to the respective absorption 𝜆_max_. The Vy_4_D_4_‐copolymer studies serve as a baseline for the current study. Prior to investigating pendant group interactions within the cyclic Vy_4_D_4_ motif, it is essential to establish that the Vy_4_D_4_ co‐oligomers/polymers support σ−σ* behavior comparable to the linear VySiOSiVy analogs [11].

**FIGURE 3:**
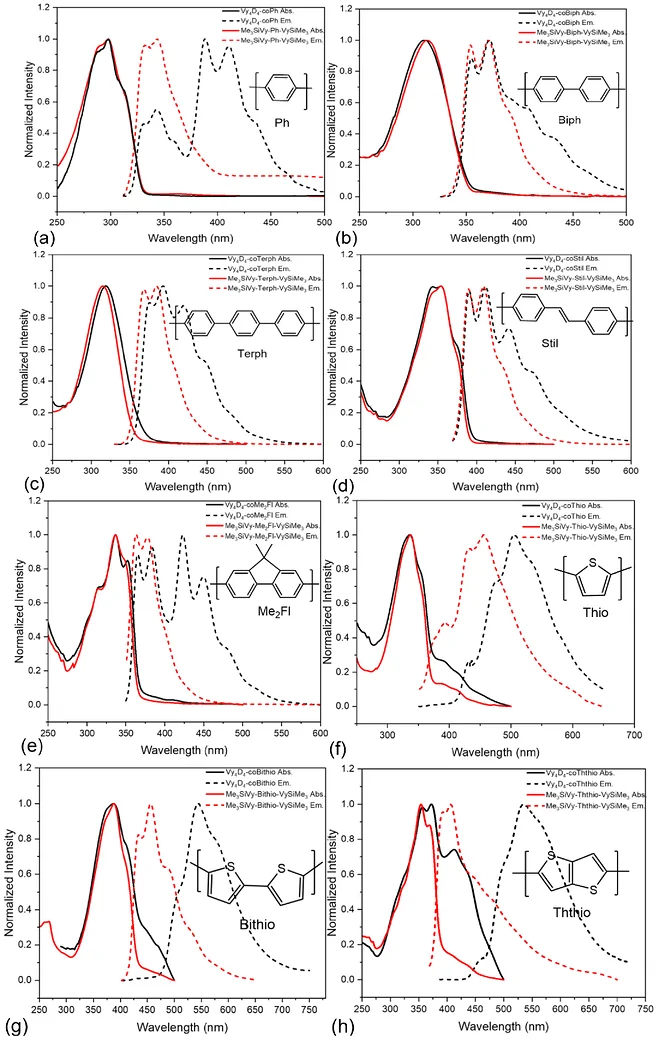
Normalized absorption (in solid lines) and emission (in dashed lines) of Vy_4_D_4_‐copolymers (in black) vs. the corresponding silane model compounds (in red). (a) Ar = benzene, (b) Ar = biphenyl, (c) Ar = terphenyl, (d) Ar = stilbene, (e) Ar = dimethylfluorene, (f) Ar = thiophene, (g) Ar = bithiophene, (h) Ar = thienothiophene.

Figure S81 compares the two kinds of model compounds. Thus, absorption 𝜆_max_ for most Vy_4_D_4_‐copolymers align closely with those of their corresponding models (Figure 3 and Table S2) in agreement with earlier studies on linear siloxane copolymer systems. In addition to these primary features, thiophene, bithiophene, thienothiophene copolymers exhibit distinct absorption shoulders at longer wavelengths and progressive red‐shifted emissions compared to the model compounds. This likely correlates with structural changes in the ground‐state to excited state Si─O─Si bond angles, shifting from smaller angles in the models to larger angles in the copolymers, resulting in more planar bond angles and improved conjugation. As noted above, a plausible explanation for the emergence of this spectral feature is a 𝜎→𝜎* electronic transition. Also, all the copolymers show enhanced Φ_F_ compared to the corresponding model compounds, indicating copolymerization of aromatic groups with cyclotetrasiloxane units enhances luminescent emission, perhaps through increased rigidity vs. models reducing propensities for raditionless decay.

As one might expect for σ−σ*, relatively weak absorptions are observed; however, the quite strong emission intensities contrast with this explanation. The exact rationale for this behavior remains unknown at this time, but likely arises from energy relaxation from σ* to a π* excited state with more efficient decay to the ground state with emission of a photon.

### 2.3 Post‐Functionalization of Vy_4_D_4_‐Copolymers with Ar′ Pendants

**SCHEME 4:**
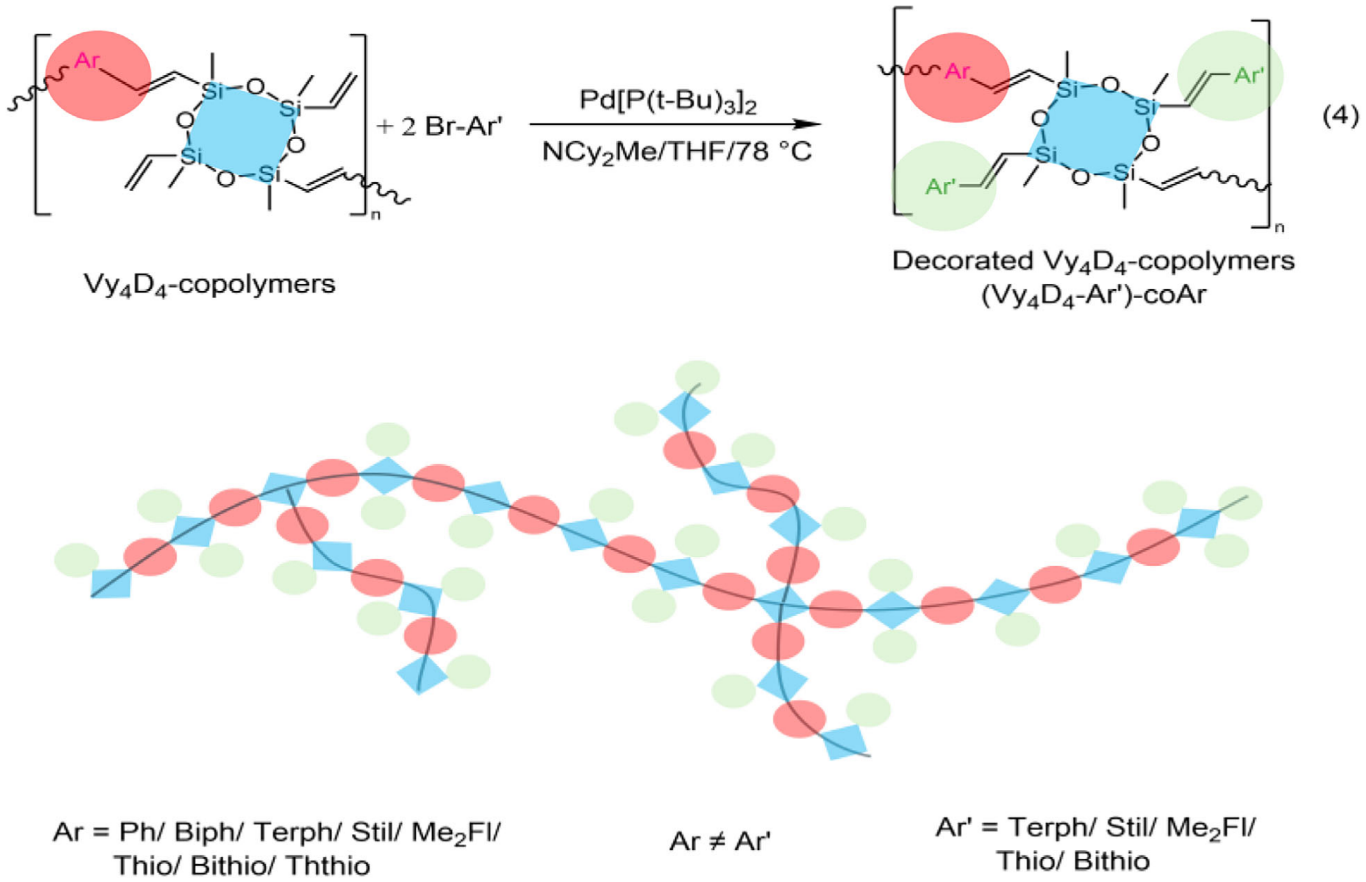
Post‐functionalization/ decoration of Vy_4_D_4_‐copolymers.

Reaction (4) in Scheme 4 illustrates the synthesis of copolymers with secondary pendant groups, “decoration.” Heck catalytic cross‐coupling was the basic synthesis method used, see Supporting Information.

Post‐functionalized (or decorated) Vy_4_D_4_ copolymers were characterized by GPC (Table S8 and Figures S18–S32), FTIR (Table S4 and Figures S38–S42), NMR (Table S9), and TGA (Table S8 and Figures S66–S80). In general, the GPC and FTIR data are consistent with the expected isolated compound structures. GPC analyses reveal decreases in DP on incorporating pendant thiophene or bithiophene groups.

TGA further shows that Td_5%/air_ decreases with increasing organic content, relative to the parent copolymers. The theoretical CYs of the post‐functionalized (decorated) Vy_4_D_4_ copolymers are calculated as two pendant Ar’ groups per repeat unit. The number of added pendants is also calculated, Table S8. On average, more than one pendant group is attached/ring. The resulting photophysical changes observed in the post‐functionalized (decorated) copolymers vs. original copolymers likely arise from introducing pendant groups.

### 2.4 Photophysical Properties of Ar'decorated Vy_4_D_4_‐Copolymers

Table 1 summarizes the steady‐state absorption and photoluminescence characteristics of the synthesized Vy_4_D_4_‐copolymers and corresponding “decorated” copolymers.

**TABLE 1:** UV–vis data and Φ_F_ for Vy_4_D_4_‐copolymers vs. post‐functionalized (decorated) Vy_4_D_4_‐copolymers.

	DP	Abs. λ_max_ (nm) ^a^	Em. λ_max_ (nm) ^b^	Stokes Shift (eV)	Φ_F_
Vy_4_D_4_‐coPh	24	297	345, 389, 411	0.99	0.03 ± 0.01
(Vy_4_D_4_ ‐Thio)‐coPh	41	297	345, 389, 411	1.16	0.01 ± 0.005
(Vy_4_D_4_ ‐Bithio)‐coPh	40	314, 353	331, 483	0.95	0.06 ± 0.02
Vy_4_D_4_‐coBiph	90	312	355, 372	0.64	0.16 ± 0.02
(Vy_4_D_4_ ‐Thio)‐coBiph	48	304	374, 395	0.76	0.09 ± 0.03
(Vy_4_D_4_ ‐Bithio)‐coBiph	35	316	480	1.34	0.07 ± 0.02
Vy_4_D_4_‐coTerph	36	318	393, 418	0.74	0.42 ± 0.01
(Vy_4_D_4_ ‐Thio)‐coTerph	32	317	417	0.94	0.16 ± 0.04
(Vy_4_D_4_ ‐Bithio)‐coTerph	15	327	482	1.22	0.08 ± 0.03
Vy_4_D_4_‐coMe_2_Fl	130	336	384, 423	0.76	0.29 ± 0.04
(Vy_4_D_4_ ‐Thio)‐coMe_2_Fl	49	337_,_ 352	424, 450	0.75	0.22 ± 0.01
(Vy_4_D_4_ ‐Bithio)‐coMe_2_Fl	9	355, 379	455, 482	0.92	0.12 ± 0.04
Vy_4_D_4_‐coThio	45	337	504	1.22	0.12 ± 0.01
(Vy_4_D_4_ ‐Terph)‐coThio	28	305	372, 401, 431, 504	1.61	0.10 ± 0.01
(Vy_4_D_4_ ‐Stil)‐coThio	32	335	509	1.27	0.08 ± 0.02
(Vy_4_D_4_ ‐Me_2_Fl)‐coThio	88	314	363, 398, 421, 531	1.62	0.04 ± 0.01
Vy_4_D_4_‐coBithio	53	388	544	0.92	0.29 ± 0.01
(Vy_4_D_4_ ‐Terph)‐coBihio	75	305	433, 463, 540	1.77	0.29 ± 0.03
(Vy_4_D_4_ ‐Stil)‐coBithio	83	334	427, 450, 541	1.42	0.14 ± 0.02
(Vy_4_D_4_ ‐Me_2_Fl)‐coBithio	161	325	424, 446, 545	1.54	0.10 ± 0.01
Vy_4_D_4_‐coThthio	55	372, 411	535	1.02	0.20 ± 0.01
(Vy_4_D_4_ ‐Me_2_Fl)‐coThthio	208	325	397, 423, 450, 539	1.52	0.11 ± 0.03

^a^ Underlined peaks are the highest intensity if multiple peaks.

^b^ Emission spectra were measured using excitation wavelengths matched to the respective absorption maxima (𝜆max).

**FIGURE 4:**
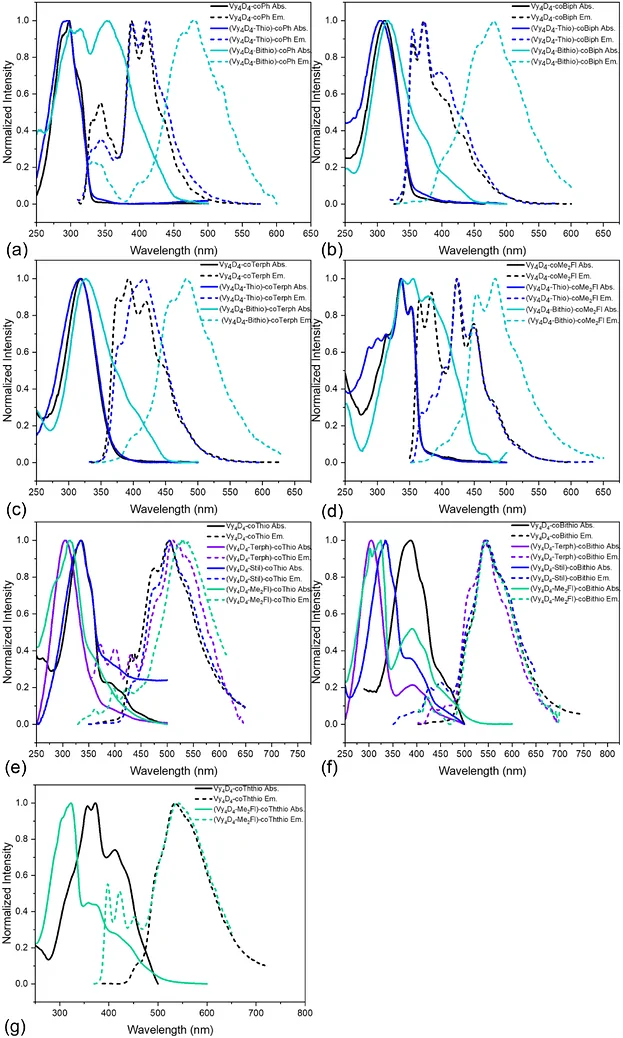
Normalized absorption (in solid lines) and emission (in dashed lines) of Vy_4_D_4_‐copolymers vs the corresponding “decorated” Vy_4_D_4_‐copolymers.

In Figure 4a–d the main chain contains aromatics (e.g., Ar = Ph/ Biph/ Terph/ Me_2_Fl), and Ar’ pendant substituent heteroaromatics (e.g., Thio/ Bithio), the resulting “decorated” copolymers exhibit distinctly different photophysical properties as evidenced by a notable red‐shifted emission λ_max_ compared to unsubstituted analogs. Three explanations are possible. In one, simple energy transfer from the main chain to the side chain heteroaromatics occurs via FRET *accessing the lowest energy excited state* in keeping with the model compounds. Alternately energy transfer occurs via σ−σ* “2‐D” conjugation around the ring. The third explanation is that both mechanisms are operative. Excited state lifetime measurements presented below suggest the latter.

For example, in Figure 4d, Vy_4_D_4_‐coMe_2_Fl/pendant thiophenes retain an absorption λ_max_ at 337 nm relative to the parent, but the original 380 nm emission λ_max_ shifts predominantly to 420 nm. Likewise, the absorption λ_max_ for Vy_4_D_4_‐coBithio/Ph, Figure 4a, broadens and redshifts to 353 nm, relative to 297 nm for the parent, with a significant λ_max_ redshift emission from 389 to 483 nm.

Coincidentally, a significant decrease in the fluorescence quantum yield is observed for the decorated copolymers compared to their parent analogs (e.g., the Φ_F_ of Vy_4_D_4_‐Thio‐coTerph drops to 0.16 from the parent's 0.42). Specifically, the reduced Φ_F_ in the decorated systems is attributed to the introduction of additional nonradiative decay pathways associated with the pendant aromatic groups. These groups can facilitate energy redistribution via Förster resonance energy transfer (FRET) and/or through extended σ–σ* conjugation around the siloxane ring, leading to population of lower‐energy excited states with increased potential for vibrational relaxation. Thus, although the decorated copolymers offer red‐shifted emission, the overall radiative efficiency is diminished.

The optical behavior of the post‐functionalized (decorated) copolymers further reflects intrinsic differences between the thiophene‐ and bithiophene‐based pendants. Bithiophene units possess inherently lower bandgaps and stronger intrachain electronic coupling than monothiophene analogues, and thus are known to exhibit more pronounced red‐shifted absorption and emission features [15].

Consistent with this, the bithiophene‐decorated polymers in this study display distinct low‐energy optical bands that originate from the intrinsic photophysics of the bithiophene pendants rather than from unintended by‐products. In contrast, the thiophene‐decorated polymers show more moderate, yet still systematic, spectral changes. These observations suggest that conjugation extends across the tetrasiloxane rings and is facilitated by electronic communication between the aromatic units in the main chain and those appended as side pendants, thereby enabling effective modulation of the copolymers’ optical properties. Again, the alternative is FRET, but see discussion on lifetimes and responses to F_4_TCNQ below.

We have previously shown that some siloxane and silsesquioxane co‐polymers exhibit multiple emissive states, indicating potential violation of Kasha's rule [2, 3]. A similar situation is observed in several of the systems studied here. In particular, the 2‐D excitation‐emission maps for Vy_4_D_4_‐coPhenyl and Vy_4_D_4_‐coDimethylfluorene (Figure S82) show fluorescence *emanating from two different origins*. In addition, time‐resolved fluorescence measurements (Figure S83) indicate that the two emissions also decay differently; the longer‐wavelength component shows a nearly exponential behavior with ∼1 ns lifetime, whereas the shorter‐wavelength emission also contains a prominent, relatively fast component, < 10 ps for Vy_4_D_4_‐coPhenyl and ∼100 ps for Vy_4_D_4_‐coDimethylfluorene. A likely explanation is a combination of FRET and σ‐σ* conjugation supporting the concept of 2‐D conjugation. Further support comes from interactions with F_4_TCNQ.

**FIGURE 5:**
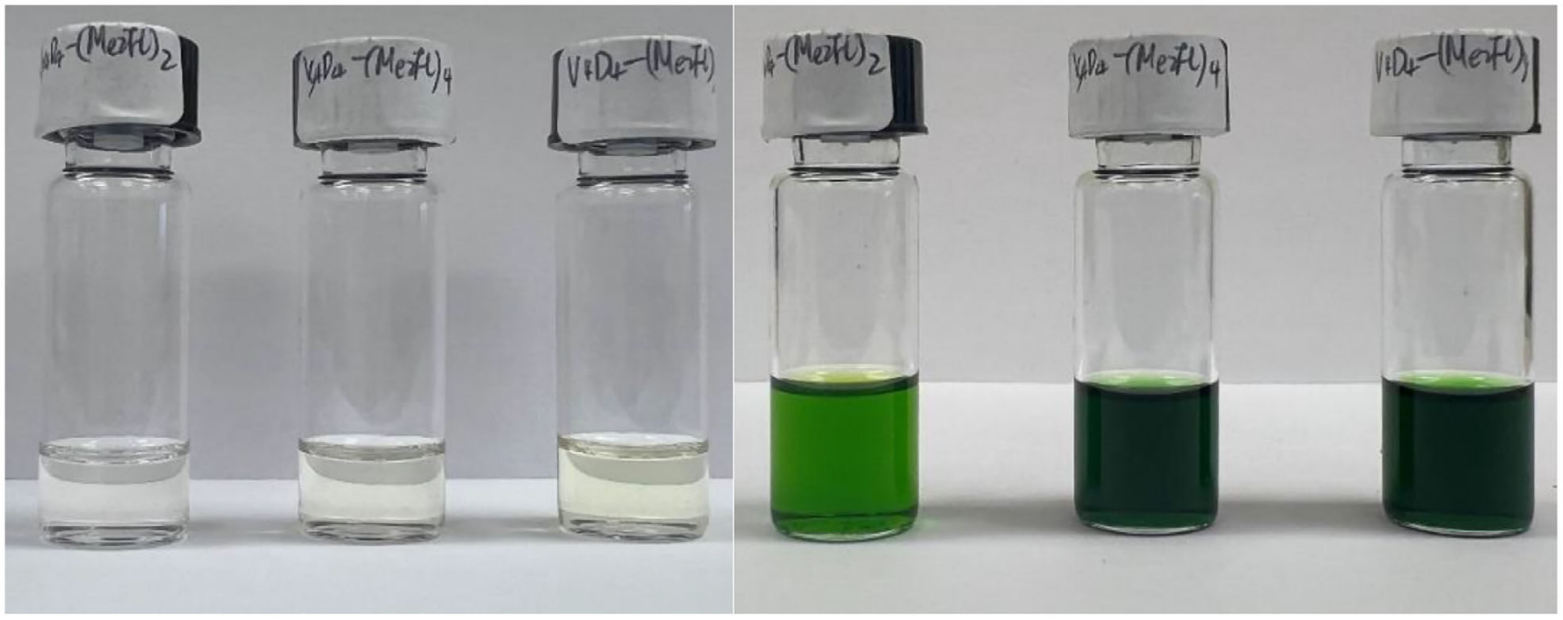
Color changes of Vy_4_D_4_‐(Me_2_Fl)_2_, Vy_4_D_4_‐(Me_2_Fl)_4_, Vy_4_D_4_‐(Me_2_Fl)_8_ solutions before (left) and after (right) doped with 50 mol% F_4_TCNQ.

**FIGURE 6:**
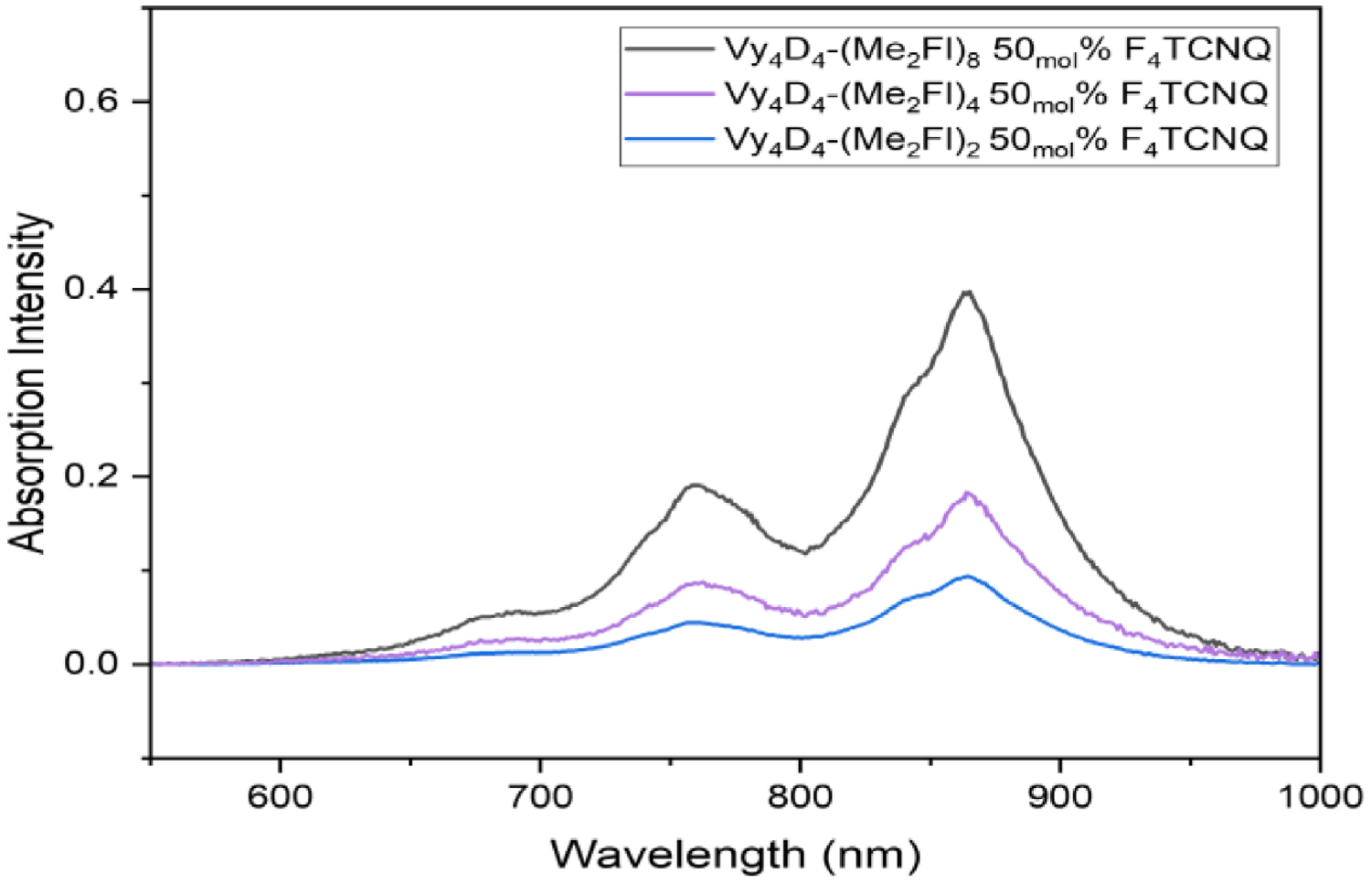
Absorption spectra for Vy_4_D_4_‐(Me_2_Fl)_2_, Vy_4_D_4_‐(Me_2_Fl)_4_, Vy_4_D_4_‐(Me_2_Fl)_8_ solutions doped with 50mol% F_4_TCNQ.

**FIGURE 7:**
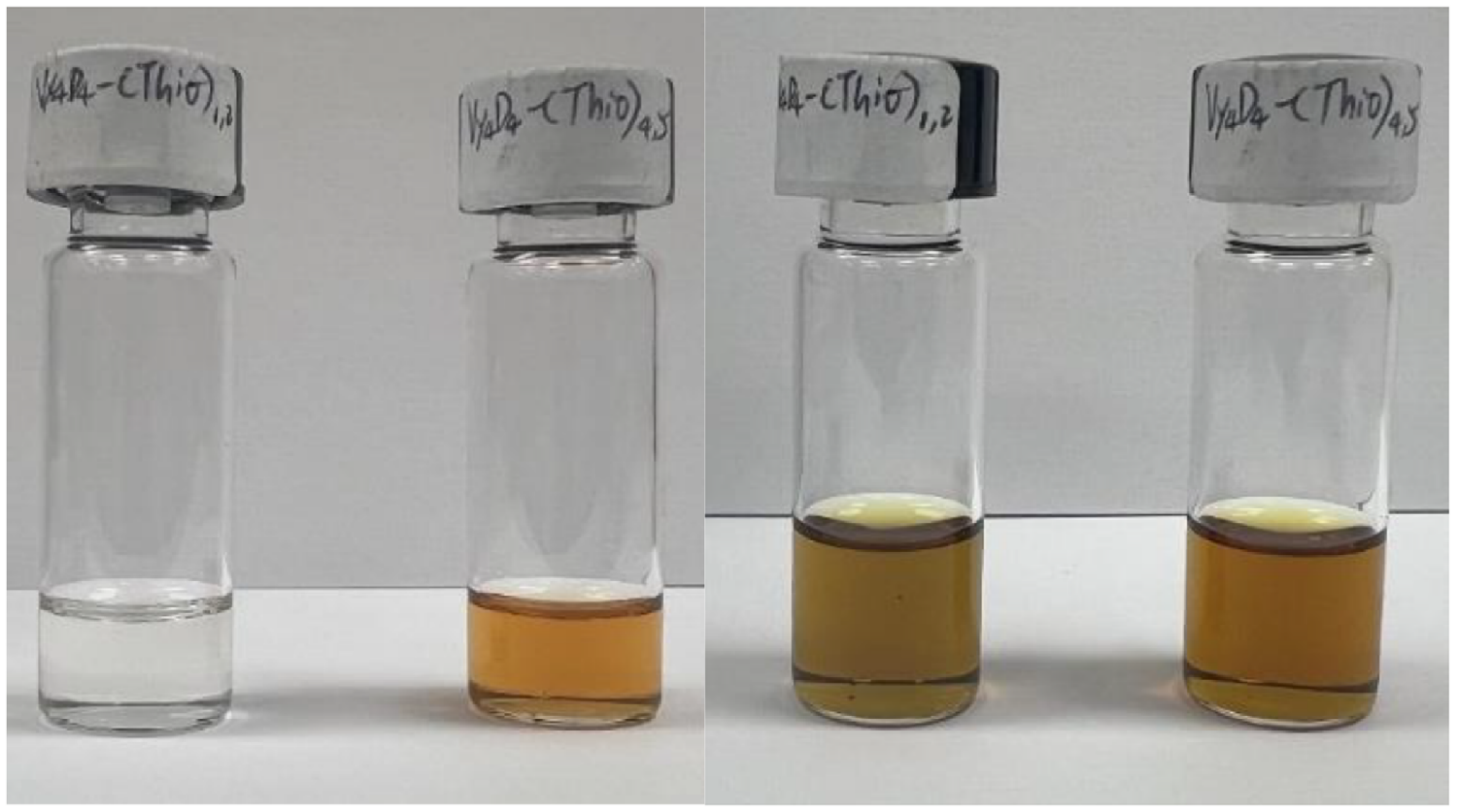
Color changes of Vy_4_D_4_‐(Thio)_1,2_, Vy_4_D_4_‐(Thio)_4,5_ solutions before (left) and after (right) doped with 50 mol% F_4_TCNQ.

**FIGURE 8:**
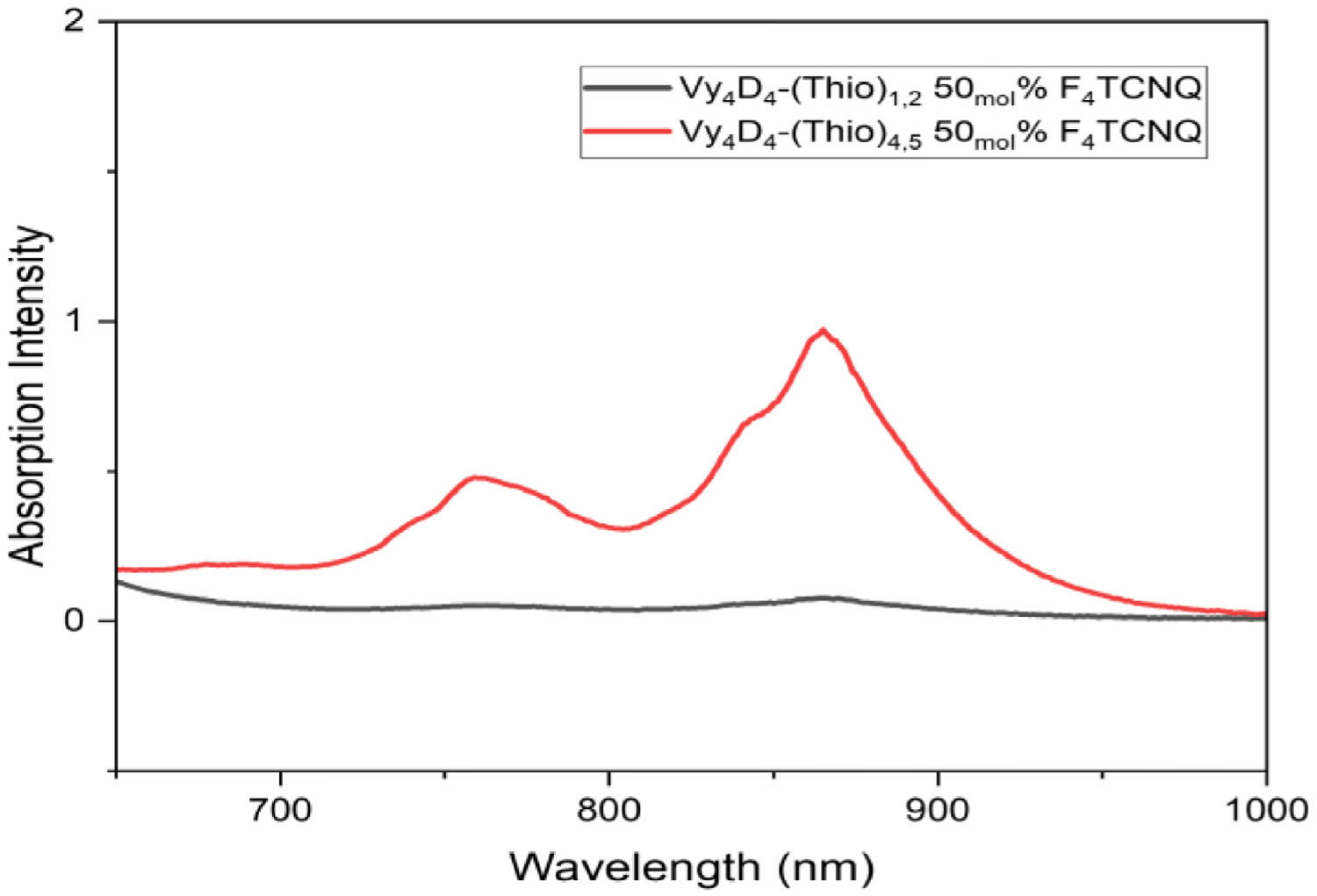
Absorption spectra for Vy_4_D_4_‐(Thio)_1,2_, Vy_4_D_4_‐(Thio)_4,5_ doped with 50 mol% F_4_TCNQ.

Likewise, we have previously reported, based on literature precedent, Si─O─Si σ−σ* conjugated system can donate electrons to F_4_TCNQ by either partial or integer charge transfer (ICT) to form the radical anion F_4_TCNQ^−^ [2, 3]. We have also previously demonstrated that model compounds do not interact with F_4_TCNQ. Thus, if Förster energy transfer were the sole mechanism operating in the present systems, then no conjugation is involved, and hence no electron transfer would be observed. However, we find that the conjugated cyclomer 1:1 copolymers exhibit electron transfer to F_4_TCNQ, and separately, we now find that decorated cyclomers *not part of copolymers* also exhibit electron transfer to F_4_TCNQ. This is amply demonstrated in Figures 5, 6, 7, 8, where the color changes seen result in the formation of F_4_TCNQ^−.^ as evidenced by its UV–vis absorptions at λ_max_ of 760 and 870 nm results not seen with model studies. Of the UV–vis spectra, only the Vy_4_D_4_‐(Thio)_1,2_ system data are much less convincing vis a vis ICT.

**FIGURE 9:**
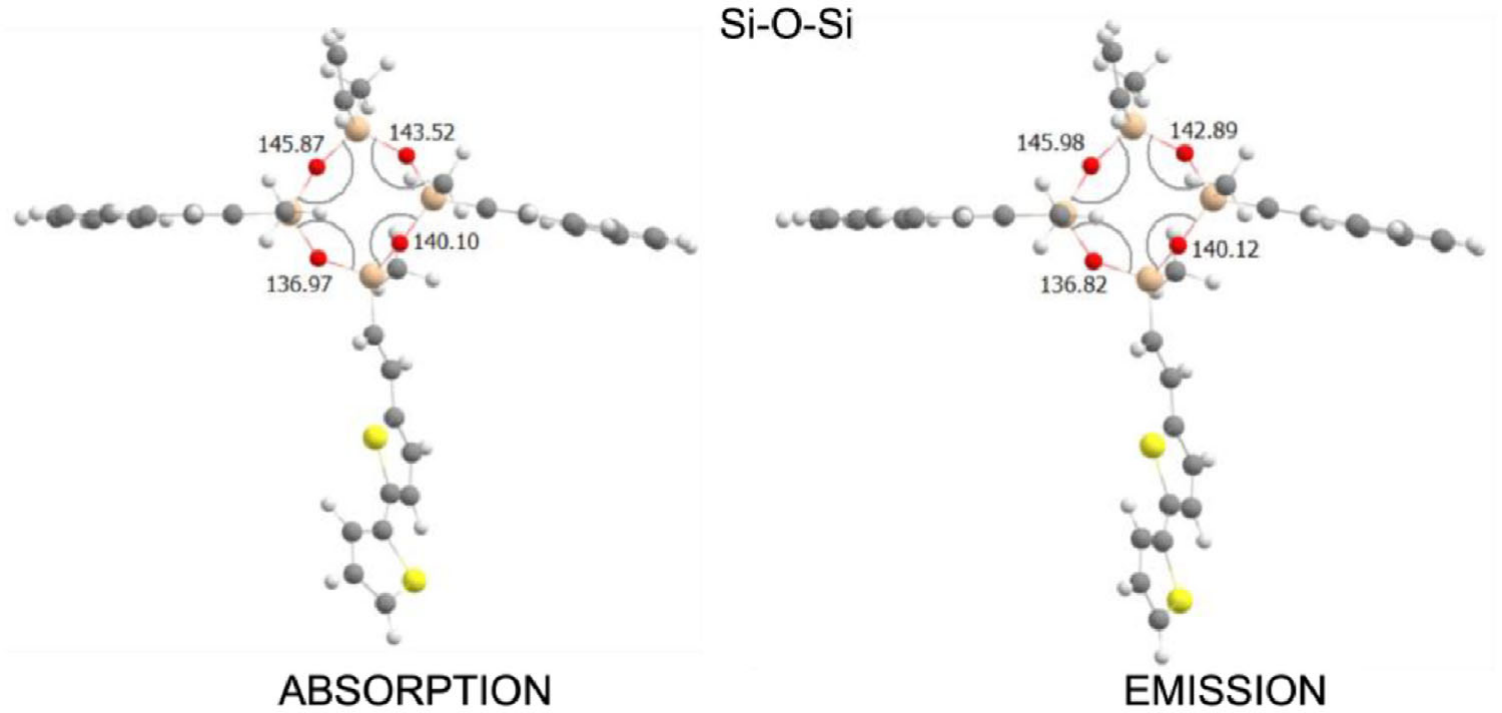
DFT derived Si─O─Si bond angles for absorption and emission conformations.

**FIGURE 10:**
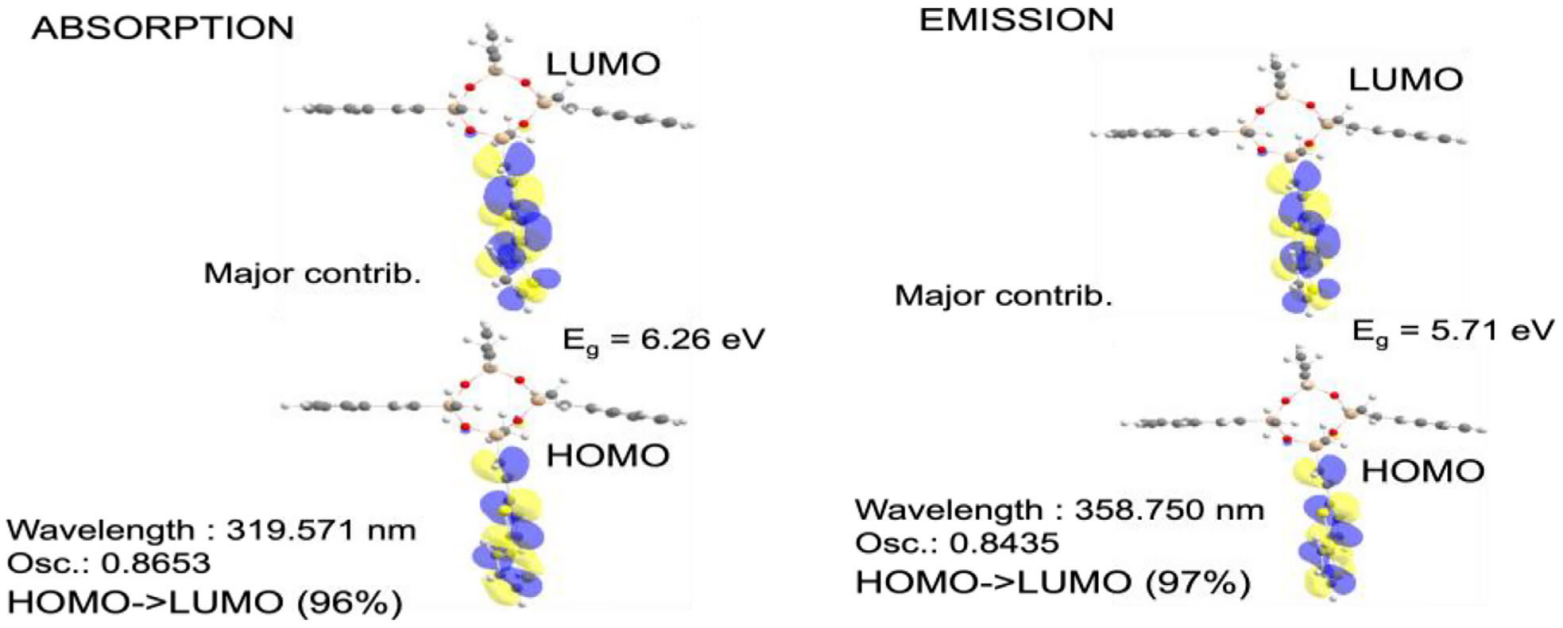
DFT calculated absorption and emission wavelengths and bandgaps.

Because the discovery of σ−σ* conjugation in these cyclomers is so unexpected, during the time this manuscript was being reviewed, we succeeded in obtaining DFT modeling of their behavior as seen in Figures 9 and 10.

**FIGURE 11:**
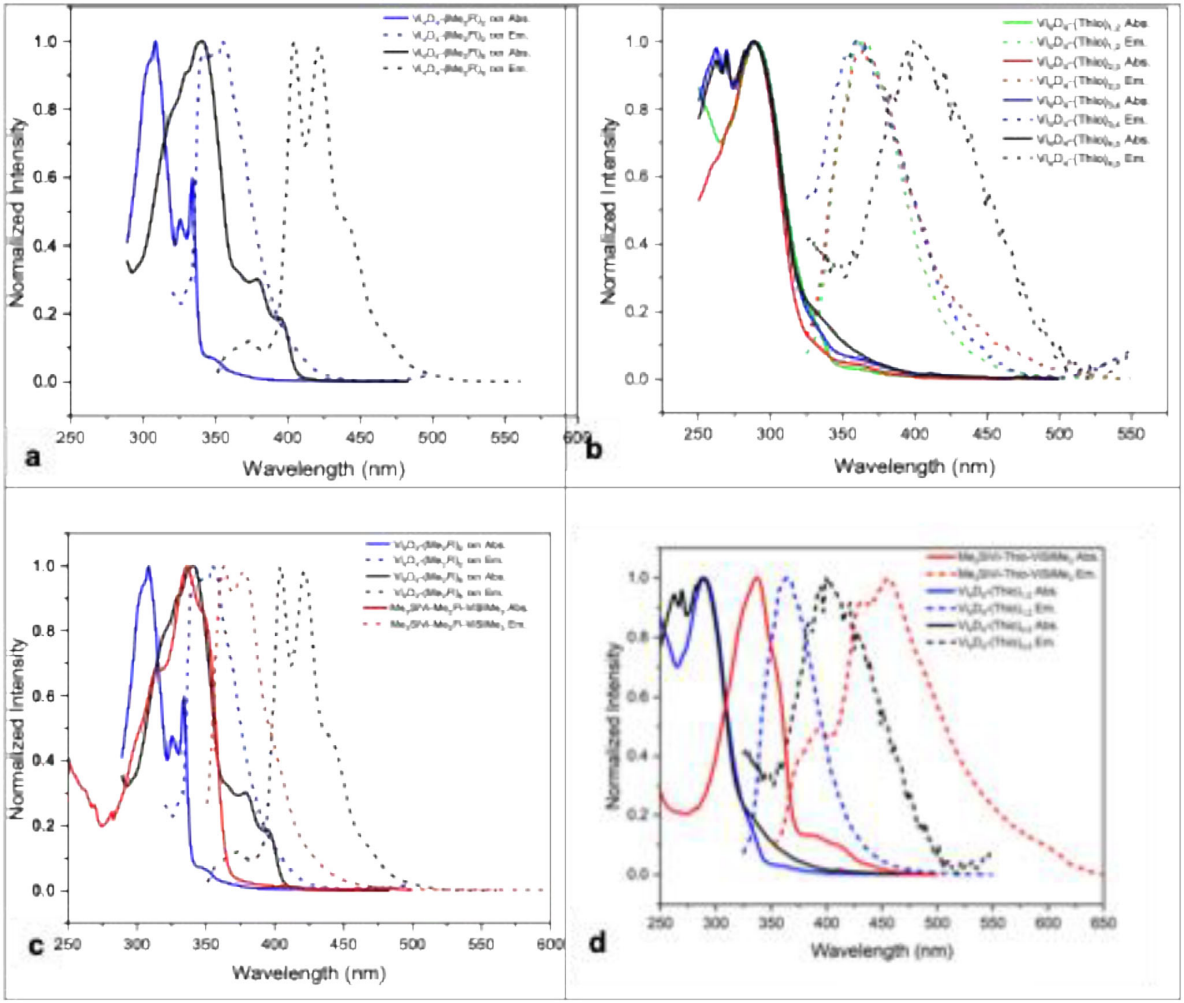
(a) Normalized absorption (solid line) and emission (dashed line) spectra for Vi_4_D_4_‐(Me_2_Fl)_8_ (black) and Vi_4_D_4_‐(Me_2_Fl)_2_ (blue). (b) Normalized absorption (solid line) and emission (dashed line) spectra for Vi_4_D_4_‐(Thio)_1,2_ (green), Vi_4_D_4_‐(Thio)_2,3_ (red), Vi_4_D_4_‐(Thio)_3,4_ (blue), and Vi_4_D_4_‐(Thio)_4,5_ (black). (c) Normalized absorption (solid line) and emission (dashed line) spectra for Vi_4_D_4_‐(Me_2_Fl)_8_ (black), Vi_4_D_4_‐(Me_2_Fl)_2_ (blue), and Me_3_SiVi‐Me_2_Fl‐ViSiMe_3_ (red). (d) Normalized absorption (solid line) and emission (dashed line) spectra for Vi_4_D_4_‐(Thio)_4,5_ (black), Vi_4_D_4_‐(Thio)_1,2_ (blue), and Me_3_SiVi‐Thio‐ViSiMe_3_ (red).

These calculations provide the background for Figure 11, absorption and emission behavior for column chromatography isolated Ar_x_Vy_4_D_4_ with various degrees “x” of substitution.

The above calculations suggest that the major HOMO→LUMO transitions for Ar_x_Vy_4_D_4_ are π‐π* based on calculated absorption and emission λ_max_; however, Figure 11c,d reveals much different behavior as “x” increases, indicating further support for cyclomeric σ−σ* conjugation.

Perhaps most revealing are the Si─O─Si bond angles averaging ≥ 140° seen in Figure 9, suggesting the ring approaches planarity as a consequence of conjugation.

## 3 Conclusions

The objective of the work reported here was to clearly define the phenomenon we have previously termed unconventional conjugation, in that we have observed photophysical properties that seem to involve conjugation across multiple Si─O─Si bonds [1, 6]. Early results suggested dπ‐pπ interactions [2]; however, a better interpretation seems to be σ‐σ* conjugation [1, 3], but the results were still somewhat tentative. In the current effort, we sought to both more succinctly define operative mechanisms but also to provide general tools for the synthesis of Si─O─Si containing copolymers easily accessible to other interested researchers. Furthermore, we formulated hypotheses of molecular structures that also might exhibit σ‐σ* conjugation as suggested in Scheme 3.

One of these structures, the Q_8_ system, suggests another route (besides T_8_ systems [9, 16]) to 3‐D σ‐σ* conjugation. The first step in exploring the potential these systems offer was to synthesize cyclomer‐aromatic copolymers and then decorated versions derived from the commercially available [MeVinylSiO]_4_ cyclomer. Photophysical studies of both types of Vy_4_D_4_‐copolymers display similar absorption 𝜆_max_ with an absorption shoulder at longer wavelengths now attributable to 𝜎→𝜎* transitions and significantly red shifted λ_max_ emissions compared with silane model compounds. The “decorated” copolymers, functionalized remotely at ring vinyls, not part of the copolymer backbone, show different photophysical behavior, suggesting the decorated groups introduce a mechanism for further modification of main chain photophysical properties and also represent a form of 2‐D conjugation.

One alternate, simple explanation to the observed photophysical properties is that the appended groups affect photophysical properties simply by Förster energy transfer rather than via conjugation. However, excited state lifetime spectroscopy reveals two competing emission mechanisms. Furthermore, studies with F_4_TCNQ doping reveal integer system charge transfer to form the radical anion, F_4_TCNQ^−^. Such a process is known for traditional conjugated organic polymers [17] and also occurs in our earlier studies^−^ [2, 3], but is not seen with model compounds.

Most importantly, we now also find ICT between F_4_TCNQ and functionalized Vy_4_D_4_ *not part of a polymer backbone*. Hence σ‐σ* conjugation appears to occur within simpler systems, meaning one only needs four Si─O─Si units for such conjugation to occur. *A new question arises: If copolymers change bond angles from model compounds at ≈110° to as much as 150°* (Figure 9) in the excited state will “decorated” cyclomers in the σ‐σ* conjugation excited state become relatively planar (as seen) and would this lead to ring currents and how would one characterize it?

The results presented above offer evidence that σ−σ* conjugation can occur (1) in simple cyclomers multiply decorated with functional groups; (2) through cyclomer‐aromatic copolymer backbones, and (3) in 2‐D by coupling the remotely decorated cyclomeric component of the cyclomer‐aromatic copolymer backbone. It seems likely that many of the systems we previously reported to offer unconventional conjugation likely involve, at least in part, σ−σ* conjugation [5, 17].

Finally, the easy syntheses and excited‐state conjugation of sets of SQ/polysiloxane‐based polymers may offer potentially new hybrid organic–inorganic semiconducting materials in OLED and photovoltaic applications. Thus, one particular motivation of the current work targets mapping structure‐property relationships to explore the potential differences that may arise as a consequence of different Si─O─Si linker sizes compared with the SQ and linear siloxane compounds we explored previously, at the same time exploring novel combinations of robust nature, long‐wavelength emission, and high Φ_f_, of potential value in flat panel display applications [18, 19, 20].

The following manuscript will demonstrate σ‐σ* conjugation in polysilazane copolymers [21].

## Conflicts of Interest

The authors declare no conflicts of interest.

## Supporting information




**Supporting File**: marc70348‐sup‐0001‐SuppMat.docx.

## Data Availability

The data that support the findings of this study are available on request from the corresponding author. The data are not publicly available due to privacy or ethical restrictions.
