# Supplementary material for: Exploring 2‐D σ−σ* Conjugation in Cyclic Polysiloxane Copolymers
Source: Macromol Rapid Commun. 2026 Jun 22;47(17):e70348. doi: 10.1002/marc.70348 (PMC13542737; doi:10.1002/marc.70348)
Supplement: Supplementary file 1 [file MARC-47-e70348-s001.docx]

Supplementary Information

**Exploring 2-D σ−σ* conjugation in cyclic polysiloxane copolymers**

Zijing Zhang,^a^ Cecilia Pilon,^b^ Aleksander Rebane,^c^ Richard M. Laine^a,d*^

^a^Deptd. of Materials Science and Engineering, ^b^ Chemistry, and ^d^ Macromolecular Science and Engineering Center, University of Michigan, Ann Arbor, MI, 48109-2136, USA

^c^Montana State University, Bozeman, MT, 59717, USA

email: [talsdad@umich.edu](mailto:talsdad@umich.edu)

**Experimental section**

**Materials**

All purchased chemicals were used as received unless otherwise indicated. Divinyltetramethyldisiloxane was purchased from ACROS ORGAMOCS (97% purity). Vinyltrimethylsilane (97% purity) was purchased from Millipore Sigma. Bis(tri-tert-butylphosphine)palladium(0) (Pd[P(t-Bu)_3_]_2_), Pd_2_(dba)_3_, N,N-dicyclohexylmethylamine (NCy_2_Me) and 1,4-dibromobenzene were purchased from TCI chemicals-Japan. 1,4-Dibromobenzene, 4,4′-dibromo-1,1′-biphenyl, 4,4″-dibromo-p-terphenyl, 2,7-Dibromo-9,9-dimethylfluorene, trans-4,4′-Dibromostilbene, 2,5-dibromothiophene, 5,5'-Dibromo-2,2'-bithiophene, 2,5-Dibromothieno[3,2-b]thiophene, bromobenzene, 4-Bromobiphenyl, 4-Bromo-p-terphenyl, 2-Bromo-9,9-dimethylfluorene, 2-bromothiophene, 5-Bromo-2,2′-bithiophene, were purchased from Sigma Aldrich. Tetrahydrofuran was dried over pre-treated molecular sieves for at least 48 h and then distilled under N_2_ from sodium/benzophenone ketyl before use.

**Synthetic Methods**

General procedure for reaction of vinyltrimethylsilane model compounds.

To an oven-dried 25 mL Schlenk flask under N_2_ were added Vinyltrimethylsilane (501 mg, 5 mmol), NCy_2_Me (0.41 g, 2.0 mmol), Br-Ar-Br (1.0 mmol). Following the addition 10 mL of THF, Pd[P(tBu)_3_]_2_ (19.4 mg, 0.04 mmol), and Pd_2_(dba)_3_ (17.3 mg, 0.02 mmol) were added. The mixture was stirred magnetically under N_2_ at 70 °C and tracked by GPC. The reaction mixture was filtered through Celite to remove Pd catalyst, and the excess Vinyltrimethylsilane was further removed by column chromatography.

General procedure for oligomerization of [MeVinylSiO]_4_ (Vy_4_D_4_) with ArBr_2_.

To a 50 mL flame-dried Schlenk flask under N_2_, a mixture of Vy_4_D_4_ (2 mmol. 382 mg), NCy_2_Me (800 mg, 4 mmol) and Br-Ar-Br (2 mmol) in 24 mL dry THF were added. Further, Pd[P(t-Bu)_3_]_2_ (54 mg, 0.1 mmol) was added. Then a rubber septum sealed condenser was added. The mixture was stirred magnetically at reflux for 2 d and then centrifuged to separate the solution and precipitate. The resulting solution was condensed by rotary evaporation and then precipitated into cold well-stirred methanol (20 mL). The mixture was centrifuged to collect the precipitate. The resulting precipitate was vacuum dried in the oven at 60 °C to give the solid or waxy product.

General procedure for decorated copolymers by addition Ar’-Br with Vy_4_D_4_-copolymers.

To a 25 mL flame-dried pressure reactor under N_2_, a mixture of Vy_4_D_4_-copolymer (0.5 mmol.), NCy_2_Me (1 mmol) and Br-Ar (1.1 mmol) in 10 mL dry THF was added. Further, Pd[P(t-Bu)_3_]_2_ (0.05mmol, 5mol%) was added, and the reactor was fully sealed. The mixture was stirred magnetically at reflux under 70 °C for 2 d. Then the reaction solution was filtered through 1 cm layer of celite and 1cm layer of silica gel to remove the remaining dissolved catalyst. Then the solution was concentrated by rotary evaporation. The concentrated solution was precipitated by adding to 20 ml cold MeOH which formed white precipitate. The precipitate was collected by filtering through filter paper and then dried in the vacuum oven at 70 °C overnight.

Removal of Pd Catalyst. To a dry 50 mL Schlenk flask under N_2_ were added the above product dissolved in THF (10 mL) and N-acetyl-L-cysteine (0.1 g) dissolved in THF (1 mL). The solution was stirred magnetically overnight at room temperature and then filtered through 1 cm Celite to remove the insoluble Pd−cysteine complex. Most solvent was removed by rotary evaporation, and the resulting solid was redissolved in a minimal amount of THF and slowly poured into cold, well-stirred methanol (20 mL) to fully precipitate the product. The product was then recovered by filtration and dried under vacuum.

Column chromatographic separations. To do so, a 20 cm long column was prepared using 25 g silica gel (230-450 mesh) and a 5:1 v:v hexane: CH_2_Cl_2_ (DCM) solution. N_2_ was then used to apply pressure to the column and the remaining solution without the dicyclohexylmethylaminehydrobromide and Pd-catalyst impurities was collected. Matrix-Assisted Laser Desorption/Time of flight Spectrometry (MALDI-Tof) was then conducted to assess the degree of functionalization on the V_4_D_4_ vinyl groups. If the ideal functionalization had been achieved, then another column was prepared to separate the product by degree of functionalization.

Before the column chromatography, thin layer chromatography (TLC) was used to determine the proper solvent combination. Solvent ratio varied by product. Elution with a 2,4,6,8, and 16:1 v:v hexane: CH_2_Cl_2_ (DCM) provided good separation of product by the degree of functionalization. Eluate was collected in fractions. A 20 cm long column was prepared using 50 g silica gel (230-450 mesh). Column chromatography allows clear separation of products as seen under 365 nm illumination.

Reaction yield calculation.

The reaction yields are calculated from the mass ratio of the final purified products against the theoretical mass expected for complete conversion of the starting monomers into the targeted disubstituted silane model compounds/co-oligomers/polymers.

**Analytical methods**

Matrix-Assisted Laser Desorption/Time of flight Spectrometry (MALDI-Tof) analyses were performed on a Bruker Autoflex Speed MALDI-Tof equipped with a 337 nm nitrogen laser in positive-ion reflection mode using 1,8,9-Trihydroxyanthracene, 97%, as matrix, and AgNO_3_ as ion source. Samples were prepared by mixing solutions of 5 parts matrix (10 mg/mL), 5 parts sample (1 mg/mL in THF), and 1 part AgNO_3_ (1 mg/mL in water). Mixtures were blotted in three different spots on a steel plate at different concentrations before analysis under high vacuum.

Fourier Transform Infrared Spectroscopy (FTIR) analyses were carried out using a Nicolet 6700 Spectrometer from Thermo Scientific in transmission mode. Approximately 4 mg of sample were mixed in a mortar with 400 mg of KBr and compressed into a sample holder. Spectra were collected for the mixtures and a background of pure KBr in the range from 400 to 4000 cm ^-1^.

Thermogravimetric analysis (TGA) analyses were run on a SDT Q600 machine from TA instruments. The analyses were carried out by heating approximately 10 mg of sample from 50° to 900 °C at 10°C/min using aluminum pans, previously cleaned by soaking in distilled water and ethanol. The carrier gas was dry air at 60 ml/min.

The TGA theoretical ceramic yield is calculated from the formula (1):

Ceramic Yield _(theoretical)_ = $\frac{4*Mw (Silica)}{Mw of the repeating unit}\times100\%$	(1)

In each repeating unit, there is one tetravinyltetramethyltetracyclosiloxane molecule. After heating in air to 1000 °C, this unit is assumed to fully convert into four moles of silica (SiO₂). So, the TGA theoretical ceramic yield is calculated from the ratio of the remaining residue to the mass before heating in each unit as in formula (1).

Gel permeation chromatography (GPC) analyses were performed in a waters 515 GPC with in-line degasser and THF as solvent. The samples were prepared by diluting approx. 1 mg of sample into 1 mL THF and filtering through PVDF filters of 0.22 microns pores. The Refraction Index detector registered all the products eluting out of the column. The estimation of molar masses is given by an internal calibration using PS standard solutions with narrow dispersity.

Nuclear Magnetic Resonance**.** All ^1^H and ^13^C NMR spectra were collected from samples dissolved in CDCl_3_ and recorded on a Varian 500 MHz spectrometer. ^1^H NMR spectra were collected at 500 MHz using a spectral width of 16 ppm, a relaxation delay of 0.5 s, a pulse angle of 45°. ^13^C NMR spectra were collected at 100 MHz using a 248 ppm spectral width, a relaxation delay of 0.1 s, a pulse angle of 45°.

Mixing F_4_TCNQ with Thio/Me_2_Fl functionalized Vy_4_D_4_ rings: To a 2 mL vial, 0.01 mmol solid Vy_4_D_4_-(Thio)_1,2_, Vy_4_D_4_-(Thio)_4,5_ , Vy_4_D_4_-(Me_2_Fl)_2,_ Vy_4_D_4_-(Me_2_Fl)_4_ or Vy_4_D_4_-(Me_2_Fl)_8_ in 1 ml DCM was added, then 0.05 mmol of F_4_TCNQ/DCM (2 mg/mL) solution was added slowly on the top of the copolymer solution. UV-vis spectra of the Vy_4_D_4_-(Thio)_n_, Vy_4_D_4_-(Me_2_Fl)_n_ mixed with 50 mol% F_4_TCNQ in DCM were measured.

TDDFT Studies used simulated spectra data along with oscillator strength (f) obtained from the M062X/6-311G**/CPCM(CH_2_Cl_2_) level of theory.

**Photophysical characterization.**

UV-vis spectrometry measurements were recorded on a Shimadzu UV-1601 UV-vis transmission spectrometer. Samples were dissolved in DCM and diluted to a concentration (10^−3^-10^−4^ M) where the absorption maximum was <10% for a 1 cm path length.

Absorbance spectra were measured in a 500-250 nm spectral range using pure DCM solvent blank correction, 5 nm band pass, 1 nm step increment, and 1 s integration time.

Photoluminescence spectra were recorded on a Fluoromax-2 fluorometer in the required solvent using excitation wavelength at the absorption $\lambda$_max_. Samples from UV-vis spectroscopy were diluted (10^−5^-10^−6^ M) to avoid excimer formation and fluorometer detector saturation.

Fluorescence spectra were measured using: 1 nm excitation band pass, 1 nm emission band pass, 1 s integration time, 1 nm emission increment, emission range Abs. λ_max_ +15nm to 650 nm, for excitation wavelength at Abs. λmax.

Photoluminescent quantum yields were measured using an integrated sphere. Samples were dissolved in CH_2_Cl_2_ (DCM) and diluted to a concentration (10^−3^-10^−4^ M) where the absorption maximum was <10% for a 1 cm path length. Absorption and emission inside the sphere were determined by comparison to a blank CH_2_Cl_2_ in cuvette (glass only). Each sample was measured three times or more.

**Tables.**

**Table S1.** GPC and TGA data for Vy_4_D_4_-copolymers.

| Cmpd | Yield  (%) | GPC | | | | TGA | | |
| --- | --- | --- | --- | --- | --- | --- | --- | --- |
|  |  | M_n_ (g/mol) | M_w_  (g/mol) | Ð | DP | Ceramic yield % | Theor yield % | T_d5%/air_ °C |
| Vy_4_D_4_ | - | 459 | 463 | 1.01 | 1 | - | - | - |
| Vy_4_D_4_-copolymers | | | | | | | | |
| -coPh | 22 | 3600 | 10000 | 2.8 | 24 | 55.0 | 57.4 | 408 |
| -coBiph | 76 | 8800 | 44400 | 5.0 | 90 | 42.8 | 48.6 | 336 |
| -coTerph | 73 | 5800 | 20700 | 3.6 | 36 | 38.3 | 42.1 | 371 |
| -coStil | 74 | 7700 | 45000 | 5.8 | 86 | 41.5 | 46.1 | 347 |
| -coMe_2_Fl | 38 | 24400 | 95300 | 3.9 | 146 | 40.6 | 44.9 | 354 |
| -coThio | 31 | 6800 | 19100 | 2.8 | 45 | 49.7 | 56.6 | 398 |
| -coBithio | 51 | 6600 | 27100 | 4.1 | 53 | 42.8 | 47.4 | 403 |
| -coThthio | 18 | 4900 | 26600 | 5.4 | 55 | 35.5 | 50.0 | 329 |

^†^GPC molecular weights determined against polystyrene standards. DP calculated from Mw.

**Table S2**. UV-vis, photoluminescent data and Φ_F_ for model compounds, Vy_4_D_4_-copolymers and decorated Vy_4_D_4_-copolymers.

|  | DP | Abs. λ_max_ (nm) | Em. λ_max_ (nm) | Stokes Shift (eV) | Φ_F_ |
| --- | --- | --- | --- | --- | --- |
| Trimethy silane model compounds [Me_3_SiVinyl-Ar-VinylSiMe_3_] | | | | | |
| -Ph- | 1 | 297 | 332, 344 | 0.57 | 0.015±0.004 |
| -Biph- | 1 | 313 | 354, 371 | 0.62 | 0.09±0.01 |
| -Terph- | 1 | 315 | 367, 385 | 0.72 | 0.10±0.01 |
| -Stil- | 1 | 354 | 390, 409 | 0.47 | 0.09±0.002 |
| -Me_2_Fl- | 1 | 336 | 364, 377 | 0.10 | 0.10±0.2 |
| -Thio- | 1 | 338 | 456 | 0.95 | 0.03±0.01 |
| -Bithio- | 1 | 387 | 457 | 0.49 | 0.11±0.01 |
| -Ththio- | 1 | 354 | 406 | 0.45 | 0.10±0.01 |
| Methoxy silane model compounds [(MeO)_2_SiMeVinyl-Ar-VinylMeSi(OMe)_2_] | | | | | |
| -Ph- ^17^ | 1 | 297 | 332, 387 | 0.44 | 0.01±0.001 |
| -Biph- ^17^ | 1 | 313 | 355, 370 | 0.61 | 0.38±0.03 |
| -Terph- ^17^ | 1 | 319 | 373, 388 | 0.56 | 0.48±0.03 |
| -Stil- ^17^ | 1 | 355 | 391, 409 | 0.46 | 0.36±0.02 |
| -Me_2_Fl- ^17^ | 1 | 336 | 382 | 0.44 | 0.30±0.01 |
| -Thio- ^17^ | 1 | 340 | 390, 430 | 0.76 | 0.01±0.001 |
| -Bithio- ^17^ | 1 | 388 | 457 | 0.45 | 0.02±0.005 |
| -Ththio- ^17^ | 1 | 354 | 406,454 | 0.45 | 0.02±0.001 |
| Vy_4_D_4_-copolymers | | | | | |
| -coPh | 24 | 297 | 345, 389, 411 | 0.99 | 0.03±0.01 |
| -coBiph | 90 | 312 | 355, 372 | 0.64 | 0.16±0.02 |
| -coTerph | 36 | 318 | 393, 418 | 0.74 | 0.42±0.01 |
| -coStil | 86 | 354 | 412, 442 | 0.49 | 0.15±0.02 |
| -coMe_2_Fl | 146 | 336 | 384, 423 | 0.76 | 0.29±0.04 |
| -coThio | 45 | 337 | 504 | 1.22 | 0.12±0.01 |
| -coBithio | 53 | 388 | 544 | 0.92 | 0.29±0.01 |
| -coThthio | 55 | 372, 411 | 535 | 1.02 | 0.20±0.01 |

^†^Underlined peaks are the highest intensity if multiple peaks. ^§^Emission spectra were measured using excitation wavelengths matched to the respective absorption maxima (𝜆_max_).

**Table S3.** MALDI-TOF, GPC and TGA data for Trimethyl silane model compounds.

| Compound | Yield  (%) | GPC | | | |
| --- | --- | --- | --- | --- | --- |
|  |  | M_n_ | M_w_ | Ð | DP |
| Vy_4_D_4_ | - | 459 | 463 | 1.01 | 1 |
| Me_3_SiVy-Thio-VySiMe_3_ | 81 | 261 | 272 | 1.04 | 1 |
| Me_3_SiVy-Bithio-VySiMe_3_ | 75 | 315 | 327 | 1.03 | 1 |
| Me_3_SiVy-Ththio-VySiMe_3_ | 78 | 335 | 388 | 1.16 | 1 |
| Me_3_SiVy-Ph-VySiMe_3_ | 45 | 292 | 303 | 1.04 | 1 |
| Me_3_SiVy-Biph-VySiMe_3_ | 39 | 348 | 360 | 1.03 | 1 |
| Me_3_SiVy-Terph-VySiMe_3_ | 32 | 414 | 427 | 1.03 | 1 |
| Me_3_SiVy-Stil-VySiMe_3_ | 50 | 412 | 424 | 1.03 | 1 |
| Me_3_SiVy-Me_2_Fl-VySiMe_3_ | 48 | 394 | 413 | 1.05 | 1 |

^†^GPC molecular weights determined against polystyrene standards. DP calculated from Mw.

**Table S4.** Representative FTIR data for selected compounds.

| **Compound** | **ν** | **Wavenumber (cm^-1^)** | **Intensity** |
| --- | --- | --- | --- |
| **Me_3_SiVi-Thio-ViSiMe_3_** | Ar C=C (bending)  Si-CH_3_  Si-CH_3_  Si-CH_3_  **Si-C**H=CH-  Si-CH_3_  Si-**CH=CH**-  Ar C=C  Si-**CH=CH**-  C-H | 735  786  838  867  971  1247  1440  1506  1592  2951 | Weak, sharp  Medium, sharp  Strong, sharp  Strong, sharp  Medium, sharp  Strong, sharp  Weak, sharp  Weak, sharp  Weak, sharp  Weak, broad |
| **Vy_4_D_4_coThio** | Ar C=C (bending)  Si-CH_3_  **Si-C**H=CH-  Si-O-Si  Si-CH_3_  Si-**CH=CH**-  Ar C=C  Si-**CH=CH**-  C-H | 740  794  974  1071  1260  1406  1513  1592  2962 | Medium, sharp  Strong, sharp  Medium, sharp  Strong, broad  Strong, sharp  Weak, sharp  Weak, sharp  Weak, sharp  Weak, broad |
| **(Vy_4_D_4_-Terph)-coThio** | Ar C=C (bending)  Si-CH_3_  Si-CH_3_  **Si-C**H=CH-  Si-O-Si  Si-CH_3_  Si-**CH=CH**-  Ar C=C  Si-**CH=CH**-  C-H | 744  787  822  960  1045  1258  1447  1483  1593  2930 | Medium, sharp  Strong, sharp  Strong, sharp  Medium, sharp  Strong, broad  Strong, sharp  Weak, sharp  Weak, sharp  Weak, sharp  Weak, broad |

**Table S5**. ^1^ H-NMR and ^13^ C-NMR peaks for Trimethyl silane model compounds.

| **Compounds** | **^1^H-NMR peaks (ppm)** | **^13^ C-NMR peaks (ppm)** |
| --- | --- | --- |
| Me_3_SiVi-Ph-ViSiMe_3_ | ^1^H NMR (500 MHz, CDCl_3_) δ 7.29 (s, 4H, Ph), 6.76 (d, *J* = 19.1 Hz, 2H, vinyl), 6.37 (d, *J* = 19.1 Hz, 2H, vinyl), 0.06 (s, 18H, methyl). | ^13^C NMR (126 MHz, CDCl_3_) δ 144.43 (Ph), 139.17 (Ph), 130.68 vinyl), 127.77 (vinyl), 0.00 (methyl). |
| Me_3_SiVi-Biph-ViSiMe_3_ | ^1^H NMR (500 MHz, CDCl_3_) δ 7.41 – 7.29 (m, 8H, Ph), 6.73 (d, *J* = 19.1 Hz, 2H, vinyl), 6.34 (d, *J* = 19.1 Hz, 2H, vinyl), 0.00 (s, 18H, methyl). | ^13^C NMR (126 MHz, CDCl_3_) δ 144.27 (Ph), 141.28 (Ph), 138.63 (Ph), 137.59 (Ph), 130.91 (vinyl), 128.17 (vinyl), 0.00 (methyl). |
| Me_3_SiVi-Terph-ViSiMe_3_ | ^1^H NMR (500 MHz, cdcl_3_) δ 7.63 – 7.51 (m, 12H, Ph), 6.92 (d, *J* = 19.2 Hz, 2H, vinyl), 6.53 (d, *J* = 19.2 Hz, 2H), 0.17 (s, 18H, methyl). | ^13^C NMR (126 MHz, cdcl_3_) δ 143.84 (Ph), 143.05 (Ph), 143.02 (Ph), 140.09 (Ph), 139.87 (Ph), 139.38 (Ph), 138.50 (Ph), 137.42 (Ph), 129.75 (vinyl), 127.23 (vinyl), -1.23 (methyl). |
| Me_3_SiVi-Stil-ViSiMe_3_ | ^1^H NMR (500 MHz, CDCl_3_) δ 7.34 – 7.24 (m, 8H, Ph), 6.93 (s, 2H, vinyl), 6.71 (d, *J* = 19.1 Hz, 2H, vinyl), 6.33 (d, *J* = 19.1 Hz, 2H, vinyl), 0.00 (s, 18H). | ^13^C NMR (126 MHz, CDCl_3_) δ 144.36 (Ph), 138.93 (Ph), 138.20 (Ph), 130.80 (vinyl), 129.40 (vinyl), 127.95 (vinyl), -0.00 (methyl). |
| Me_3_SiVi-Me_2_Fl-ViSiMe_3_ | ^1^H NMR (500 MHz, CDCl_3_) δ 7.45 – 7.17 (m, 6H, Ph), 6.78 (d, *J* = 19.3 Hz, 2H, vinyl), 6.35 (d, *J* = 19.1 Hz, 2H, vinyl), 1.31 (s, 6H, methyl on fluorene), 0.00 (s, 18H, methyl on silane). | ^13^C NMR (126 MHz, CDCl_3_) δ 145.07 (Ph), 140.01 (Ph), 138.69 (Ph), 138.25 (Ph), 137.86 (Ph), 129.90 (vinyl), 127.04 (vinyl), 47.76 (quaternary carbon), 28.30 (methyl), 0.00 (methyl). |
| Me_3_SiVi-Thio-ViSiMe_3_ | ^1^H NMR (500 MHz, CDCl_3_) δ 7.06 (d, *J* = 3.9 Hz, 2H, thiophene), 6.97 (d, *J* = 10.0 Hz, 2H, vinyl), 6.54 (d, *J* = 18.8 Hz, 2H, vinyl), 0.17 (s, 18H, methyl). | ^13^C NMR (126 MHz, CDCl_3_) δ 136.16 (thiophene), 135.43 (thiophene), 126.18 (vinyl), 113.61 (vinyl), -1.35 (methyl). |
| Me_3_SiVi-Bithio-ViSiMe_3_ | ^1^H NMR (500 MHz, CDCl_3_) δ 7.03 (d, *J* = 3.6 Hz, 2H, thiophene), 6.88 (d, *J* = 18.6 Hz, 2H, thiophene), 6.85 (d, *J* = 4.0 Hz, 2H, vinyl), 6.18 (d, *J* = 18.8 Hz, 2H, vinyl), 0.14 (s, 18H, methyl). | ^13^C NMR (126 MHz, CDCl_3_) δ 136.07 (thiophene), 131.17 (thiophene), 129.88 (thiophene), 127.03 (thiophene), 123.98 (vinyl), 113.57 (vinyl), -1.14 (methyl). |
| Me_3_SiVi-Ththio-ViSiMe_3_ | ^1^H NMR (500 MHz, CDCl_3_) δ 7.05 (s, 2H, thienothiophene), 6.95 (d, *J* = 18.8 Hz, 2H, vinyl), 6.22 (d, *J* = 18.8 Hz, 2H, vinyl), 0.15 (s, 18H, methyl). | ^13^C NMR (126 MHz, CDCl_3_) δ 136.88 (thiophene), 130.72 (thiophene), 130.06 (thiophene), 118.55 (vinyl), 113.65 (vinyl), -1.12 (methyl). |

**Table S6**. ^1^ H-NMR peaks for Vy_4_D_4_-copolymers.

| **Copolymers** | **^1^H-NMR peaks (ppm)** |
| --- | --- |
| Vy_4_D_4_-coPh | ^1^H NMR (500 MHz, cdcl_3_) δ 7.40 (s, 4H, phenyl), 7.04 (s, 2H, vinyl), 6.32 (s, 2H, vinyl), 6.10 – 5.93 (m, 4H, vinyl), 5.91 – 5.78 (m, 2H, vinyl), 0.32 – 0.15 (m, 12H, methyl). |
| Vy_4_D_4_-coBiph | ^1^H NMR (500 MHz, cdcl_3_) δ 7.61 – 7.29 (m, 8H, phenyl), 7.17 – 7.01 (m, 2H, vinyl), 6.49 – 6.26 (m, 2H, vinyl), 6.24 – 5.93 (m, 4H, vinyl), 5.97 – 5.77 (m, 2H, vinyl), 0.57 – 0.07 (m, 12H, methyl). |
| Vy_4_D_4_-coTerph | ^1^H NMR (500 MHz, cdcl_3_) δ 7.70 – 7.18 (m, 12H, phenyl), 7.17 – 7.02 (m, 2H, vinyl), 6.61 – 6.24 (m, 2H, vinyl), 6.22 – 5.95 (m, 4H, vinyl), 5.95 – 5.59 (m, 2H, vinyl), 0.73 – 0.17 (m, 12H, methyl). |
| Vy_4_D_4_-coStil | ^1^H NMR (500 MHz, cdcl_3_) δ 7.5 – 7.28 (m, 8H, phenyl), 7.16 – 7.01 (m, 4H, vinyl), 6.52 – 6.18 (m, 2H, vinyl), 6.16 – 5.95 (m, 4H, vinyl), 5.93 – 5.75 (m, 2H, vinyl), 0.70 – 0.17m, 14H, methyl). |
| Vy_4_D_4_-coMe_2_Fl | ^1^H NMR (500 MHz, cdcl_3_) δ 7.75 – 7.30 (m, 6H, phenyl), 7.24 – 7.02 (m, 2H, vinyl), 6.57 – 6.29 (m, 2H, vinyl), 6.28 – 5.94 (m, 4H, vinyl), 5.96 – 5.74 (m, 2H, vinyl), 1.55 – 1.38 (m, 6H, methyl on fluorene), 0.47 – 0.06 (m, 12H, methyl on tetrasiloxane). |
| Vy_4_D_4_-coThio | ^1^H NMR (500 MHz, cdcl_3_) δ 7.18 – 6.94 (m, 2H, thiophene), 6.92 – 6.69 (m, 2H, vinyl), 6.19 – 5.93 (m, 6H, vinyl), 5.92 – 5.76 (m, 2H, vinyl), 0.29 – 0.16 (m, 12H, methyl). |
| Vy_4_D_4_-coBithio | ^1^H NMR (500 MHz, cdcl_3_) δ 7.15 – 6.95 (m, 4H, thiophene), 6.95 – 6.81 (m, 2H, vinyl), 6.15 – 5.93 (m, 6H, vinyl), 5.91 – 5.76 (m, 2H, vinyl), 0.32 – 0.17 (m, 12H, methyl). |
| Vy_4_D_4_-coThthio | ^1^H NMR (500 MHz, cdcl_3_) δ 7.41 – 7.07 (m, 2H, thiophene), 7.06 – 6.62 (m, 2H, vinyl), 6.31 – 5.92 (m, 6H, vinyl), 5.89 – 5.71 (m, 2H, vinyl), 0.32 – 0.08 (m, 12H, methyl). |

**Table S7**. ^13^C NMR peaks for Vy_4_D_4_-copolymers.

| **Copolymers** | **^13^C NMR peaks (ppm)** |
| --- | --- |
| Vy_4_D_4_-coPh | ^13^C NMR (126 MHz, CDCl_3_) δ 145.53 (vinyl), 138.12 (vinyl), 133.70 (vinyl), 127.04 (phenyl), 125.48 (phenyl), -0.16 (methyl), -0.68 (methyl). |
| Vy_4_D_4_-coBiph | ^13^C NMR (126 MHz, cdcl_3_) δ 145.54 (vinyl), 140.67 (vinyl), 137.16 (vinyl), 136.46 (vinyl), 133.73(vinyl), 127.32 (phenyl), 125.45 (phneyl), -0.09 (methyl), -0.55 (methyl). |
| Vy_4_D_4_-coTerph | ^13^C NMR (126 MHz, CDCl_3_) δ 145.50 (vinyl), 140.70 (vinyl), 139.83 (vinyl), 137.13 (vinyl), 136.32 (vinyl), 133.73 (vinyl), 127.36 (phenyl), 125.43 (phenyl), -0.14 (methyl), -0.68 (methyl). |
| Vy_4_D_4_-coStil | ^13^C NMR (126 MHz, CDCl_3_) δ 145.58 (vinyl), 137.40 (vinyl), 136.44 (vinyl), 133.70 (vinyl), 128.56 (phenyl), 127.24 (phenyl), 126.88 (phenyl), 125.21, -0.13 (methyl). |
| Vy_4_D_4_-coThio | ^13^C NMR (126 MHz, CDCl_3_) δ 138.61 (vinyl), 136.11 (vinyl), 133.87 (vinyl), 130.13 (vinyl), 127.44 (thiophene), 126.71 (thiophene), 125.53 (thiophene), 113.98 (thiophene), -0.68 (methyl), -3.37 (methyl). |
| Vy_4_D_4_-coBithio | ^3^C NMR (126 MHz, CDCl_3_) δ 138.15 (vinyl), 136.26 (vinyl), 133.83 (vinyl), 129.84 (thiophene), 127.83 (thiophene), 127.04 (thiophene), 124.38 (thiophene), 122.82 (thiophene), 113.68 (thiophene), -0.16, -0.68 (methyl), -3.42 (methyl). |
| Vy_4_D_4_-coThthio | ^13^C NMR (126 MHz, CDCl_3_) δ 138.92 (vinyl), 136.19 (vinyl), 133.82 (vinyl), 124.98 (thiophene), 122.78 (thiophene), 119.54 (thiophene), 116.06 (thiophene), -0.71 (methyl), -3.42 (methyl). |

**Table S8.** GPC and TGA data for decorated Vy_4_D_4_-copolymers.

| Compound | Yield  (%) | GPC | | | | TGA | | | |
| --- | --- | --- | --- | --- | --- | --- | --- | --- | --- |
|  |  | M_n_ | M_w_ | Ð | DP | Ceramic yield % | Theor yield % | T_d5%/air_ °C | No. of  pendants |
| (Vy_4_D_4_ -Thio)-coPh | 77 | 9200 | 24000 | 2.6 | 41 | 44.2 | 41.1 | 334 | 1.5 |
| (Vy_4_D_4_ -Thio)-coBiph | 69 | 7900 | 31600 | 4.0 | 48 | 38.3 | 36.5 | 333 | 1.6 |
| (Vy_4_D_4_ -Thio)-coTerph | 78 | 5600 | 23300 | 4.1 | 32 | 31.5 | 32.6 | 299 | 2.3 |
| (Vy_4_D_4_ -Thio)-coMe_2_Fl | 37 | 12000 | 34600 | 2.9 | 49 | 35.0 | 34.4 | 324 | 1.8 |
| (Vy_4_D_4_ -Bithio)-coPh | 59 | 7900 | 29800 | 3.8 | 40 | 35.5 | 32.1 | 297 | 1.6 |
| (Vy_4_D_4_ -Bithio)-coBiph | 90 | 7800 | 28600 | 3.7 | 35 | 30.8 | 29.1 | 252 | 1.7 |
| (Vy_4_D_4_ -Bithio)-coTerph | 72 | 4100 | 13100 | 3.2 | 15 | 24.0 | 26.7 | 240 | 2.6 |
| (Vy_4_D_4_ -Bithio)-coMe_2_Fl | 89 | 4700 | 7600 | 1.6 | 9 | 28.9 | 27.8 | 274 | 1.8 |
| (Vy_4_D_4_ -Terph)-coThio | 73 | 10800 | 25000 | 2.3 | 28 | 21.6 | 27.2 | 256 | 3.0 |
| (Vy_4_D_4_ -Terph)-coBithio | 64 | 7100 | 72200 | 10.2 | 75 | 20.0 | 24.9 | 323 | 3.0 |
| (Vy_4_D_4_ -Stil)-coThio | 53 | 12300 | 24800 | 2.0 | 32 | 26.5 | 30.7 | 339 | 2.7 |
| (Vy_4_D_4_ -Stil)-coBithio | 62 | 7100 | 72200 | 10.2 | 83 | 24.1 | 27.8 | 283 | 2.7 |
| (Vy_4_D_4_ -Me_2_Fl)-coThio | 59 | 3300 | 71000 | 21.8 | 88 | 20.0 | 29.7 | 245 | 4.0 |
| (Vy_4_D_4_ -Me_2_Fl)-coBithio | 81 | 10500 | 143800 | 13.7 | 161 | 23.6 | 26.9 | 327 | 2.7 |
| (Vy_4_D_4_ -Me_2_Fl)-coThthio | 75 | 7100 | 180400 | 25.4 | 208 | 24.8 | 27.7 | 355 | 2.5 |

^†^GPC molecular weights determined against polystyrene standards. DP calculated from Mw.

**Table S9**. ^1^H and ^13^C NMR peaks for decorated Vy_4_D_4_-copolymers.

| **Decorated Copolymers** | **^1^H-NMR peaks (ppm)** | **^13^ C-NMR peaks (ppm)** |
| --- | --- | --- |
| (Vy_4_D_4_-Thio)-coPh | ^1^H NMR (500 MHz, cdcl_3_) δ 7.35 (s, 4H), 7.23 – 7.08 (m, 9H), 7.07 – 7.01 (m, 2H), 7.00 – 6.78 (m, 4H), 6.48 – 6.21 (m, 2H), 6.20 – 5.94 (m, 3H), 5.93 – 5.80 (m, 1H), 0.31 (s, 12H). | ^13^C NMR (126 MHz, CDCl_3_) δ 145.76, 144.71, 138.69, 127.70, 127.12, 126.89, 125.61, 124.62, 0.00. |
| (Vy_4_D_4_-Bithio)-coPh | ^1^H NMR (500 MHz, cdcl_3_) δ 7.49 – 7.27 (m, 4H), 7.24 – 7.14 (m, 2H), 7.12 – 7.05 (m, 3H), 6.99 – 6.84 (m, 4H), 6.48 – 6.15 (m, 3H), 6.07 – 5.98 (m, 2H), 5.92 – 5.86 (m, 2H), 0.35 – 0.29 (m, 12H). | ^13^C NMR (126 MHz, CDCl_3_) δ 137.47, 137.07, 136.33, 135.88, 129.78, 127.93, 127.86, 126.97, 126.79, 125.84, 125.01, 124.79, 124.59, 124.48, 124.40, 124.26, 124.01, 123.83, 123.78, 123.75, 122.99, 115.01, 113.31, 77.28, 77.03, 76.77, 1.05. |
| (Vy_4_D_4_-Thio)-coBiph | ^1^H NMR (500 MHz, cdcl_3_) δ 7.79 – 7.31 (m, 8H), 7.28 – 7.03 (m, 7H), 7.03 – 6.77 (m, 2H), 6.52 – 6.21 (m, 3H), 6.20 – 5.97 (m, 3H), 5.96 – 5.86 (m, 0H), 0.34 (s, 11H). | ^13^C NMR (126 MHz, CDCl_3_) δ 145.67, 144.74, 140.69, 138.67, 138.51, 137.13, 136.37, 133.85, 127.68, 127.38, 127.20, 126.86, 125.59, 124.68, 77.39, 1.19. |
| (Vy_4_D_4_-Bithio)-coBiph | ^1^H NMR (500 MHz, cdcl_3_) δ 7.82 – 7.31 (m, 8H), 7.25 – 7.15 (m, 1H), 7.11 – 7.06 (m, 1H), 6.51 – 6.25 (m, 3H), 6.20 – 6.00 (m, 3H), 5.99 – 5.80 (m, 1H), 0.36 (s, 14H), 7.05 – 6.93 (m, 2H), 0.34-0.36 (m. 12H) | ^13^C NMR (126 MHz, CDCl_3_) δ 144.49, 137.21, 136.03, 135.30, 134.84, 129.11, 128.74, 126.89, 126.82, 126.17, 125.76, 123.55, 123.45, 123.36, 123.22, 122.90, 122.79, 122.74, 122.72, 121.64, 112.27, 76.25, 76.00, 75.75, 0.00. |
| (Vy_4_D_4_-Thio)-coTerph | ^1^H NMR (500 MHz, cdcl_3_) δ 7.90 – 7.29 (m, 12H), 7.25 – 7.05 (m, 7H), 7.05 – 6.76 (m, 4H), 6.54 – 6.25 (m, 2H), 6.23 – 5.97 (m, 2H), 0.73 – 0.28 (m, 12H). | ^13^C NMR (126 MHz, CDCl_3_) δ 145.71, 144.73, 138.70, 137.56, 137.07, 129.25, 127.93, 127.70, 127.43, 127.24, 126.91, 125.64, 125.58, 124.73, 124.61, 124.52, 123.93, 116.37, 113.31, 0.00. |
| (Vy_4_D_4_-Bithio)-coTerph | ^1^H NMR (500 MHz, cdcl_3_) δ 7.86 – 7.27 (m, 12H), 7.24 – 7.15 (m, 8H), 7.10 – 7.01 (m, 9H), 6.99 – 6.89 (m, 3H), 6.52 – 6.29 (m, 1H), 6.18 – 5.99 (m, 2H), 0.55 – 0.16 (m, 12H). | ^13^C NMR (126 MHz, CDCl_3_) δ 137.06, 136.33, 135.87, 130.14, 127.93, 127.88, 127.77, 126.79, 124.88, 124.59, 124.48, 124.40, 124.25, 124.01, 123.93, 123.82, 123.77, 123.63, 122.67, 122.21, 118.31, 113.30, -0.25. |
| (Vy_4_D_4_-Thio)-coMe_2_Fl | ^1^H NMR (500 MHz, cdcl_3_) δ 7.98 – 7.32 (m, 2H), 7.22 – 7.02 (m, 10H), 7.00 – 6.75 (m, 2H), 6.57 – 6.23 (m, 1H), 6.22 – 5.98 (m, 2H), 5.96 – 5.77 (m, 1H), 1.53 – 1.31 (m, 8H), 0.72 – 0.29 (m, 12H). | ^13^C NMR (126 MHz, CDCl_3_) δ 146.57, 139.49, 138.65, 137.33, 127.70, 127.46, 126.85, 126.42, 125.93, 125.61, 124.47, 120.86, 120.36, 113.40, 77.38, 68.15, 46.85, 27.27, -0.00. |
| (Vy_4_D_4_-Terph)-coThio | ^1^H NMR (500 MHz, cdcl_3_) δ 7.90 – 7.30 (m, 35H), 7.20 – 6.91 (m, 5H), 6.89 – 6.56 (m, 2H), 6.50 – 6.21 (m, 2H), 6.20 – 5.90 (m, 2H), 0.50 – 0.12 (m, 12H). | ^13^C NMR (126 MHz, CDCl_3_) δ 145.63, 140.58, 140.15, 139.40, 138.55, 136.81, 130.36, 128.86, 128.84, 128.27, 127.40, 127.33, 127.06, 126.99, 126.47, 125.02, -0.22. |
| (Vy_4_D_4_-Stil)-coThio | ^1^H NMR (500 MHz, cdcl_3_) δ 7.70 – 7.28 (m, 17H), 7.16 – 6.61 (m, 12H), 6.50 – 6.25 (m, 1H), 6.23 – 5.81 (m, 2H), 0.58 – 0.09 (m, 12H). | ^13^C NMR (126 MHz, CDCl_3_) δ 145.81, 144.48, 140.82, 138.71, 137.62, 137.40, 130.37, 129.02, 128.89, 128.88, 128.83, 128.36, 128.32, 127.84, 127.28, 127.18, 126.88, 126.73, 126.53, 126.35, 124.93, 0.00. |
| (Vy_4_D_4_-Me_2_Fl)-coThio | ^1^H NMR (500 MHz, cdcl_3_) δ 7.80 – 7.27 (m, 15H), 7.21 – 6.98 (m, 15H), 6.89 – 6.36 (m, 3H), 6.29 – 5.96 (m, 1H), 5.93 – 5.67 (m, 0H), 1.58 – 0.92 (m, 36H), 0.23 – 0.04 (m, 12H). | ^13^C NMR (126 MHz, CDCl_3_) δ 153.70, 152.94, 152.51, 149.64, 139.75, 137.92, 137.84, 137.01, 136.17, 135.83, 135.69, 128.90, 127.52, 127.46, 126.45, 126.24, 125.97, 124.84, 123.52, 121.66, 121.57, 119.88, 119.36, 119.21, 119.01, 118.64, 118.51, 112.53, 112.16, 45.80, 26.04, -0.00. |
| (Vy_4_D_4_-Terph)-coBithio | ^1^H NMR (500 MHz, cdcl_3_) δ 7.89 – 7.35 (m, 35H), 7.22 – 6.63 (m, 6H), 6.53 – 6.20 (m, 1H), 6.18 – 5.83 (m, 1H), 0.63 – 0.01 (m, 12H). | ^13^C NMR (126 MHz, CDCl_3_) δ 140.22, 136.40, 128.84, 127.54, 127.39, 127.30, 127.11, 127.05, 126.83, 126.71, 113.95, 0.00. |
| (Vy_4_D_4_-Stil)-coBithio | ^1^H NMR (500 MHz, cdcl_3_) δ 7.66 – 7.29 (m, 16H), 7.17 – 6.73 (m, 13H), 6.46 – 6.21 (m, 1H), 6.14 – 5.91 (m, 1H), 0.55 – 0.11 (m, 12H). | ^13^C NMR (126 MHz, CDCl_3_) δ 149.32, 145.65, 140.66, 137.32, 137.23, 136.91, 136.47, 128.86, 128.73, 128.71, 128.67, 128.29, 128.24, 128.18, 127.70, 127.66, 127.12, 126.90, 126.72, 126.69, 126.57, 126.56, 126.53, 126.36, 124.39, 114.14, 113.76, 77.30, 77.04, 76.79, -0.23. |
| (Vy_4_D_4_-Me_2_Fl)-coBithio | ^1^H NMR (500 MHz, cdcl_3_) δ 7.78 – 7.27 (m, 18H), 6.56 – 6.24 (m, 2H), 1.47 – 1.29 (m, 19H), 0.39 – 0.20 (m, 14H), 7.25 – 7.05 (m, 4H), 7.01 – 6.75 (m, 14H), 6.17 – 5.96 (m, 4H), 1.47 – 1.29 (m, 19H), 0.39 – 0.20 (m, 12H) | ^13^C NMR (126 MHz, CDCl_3_) δ 154.17, 153.43, 146.66, 143.67, 139.92, 139.09, 139.02, 138.90, 138.39, 137.17, 136.87, 128.70, 127.98, 127.49, 127.19, 127.15, 126.29, 126.02, 124.80, 124.45, 122.74, 120.92, 120.83, 120.55, 120.40, 120.25, 120.13, 119.65, 77.43, 77.18, 76.93, 46.89, 27.17, 0.04. |
| (Vy_4_D_4_-Me_2_Fl)-coThthio | ^1^H NMR (500 MHz, cdcl_3_) δ 7.64 (s, 1H), 7.29 – 6.65 (m, 29H), 6.35 (s, 1H), 6.03 (s, 2H), 1.41 (s, 19H), 0.29 (s, 12H). | ^13^C NMR (126 MHz, CDCl_3_) δ 154.00, 153.57, 150.69, 146.58, 140.81, 139.82, 138.97, 138.90, 138.76, 137.22, 136.95, 128.57, 127.50, 127.29, 127.02, 126.17, 125.88, 125.54, 122.63, 120.69, 119.69, 113.58, 113.22, 46.86, 27.10, -0.09. |

**Figures.**

**Figure S1**. GPC for Vy_4_D_4_.

**Figure S2**. GPC for Me_3_SiVy-Ph-VySiMe_3_.

**Figure S3**. GPC for Me_3_SiVy-Biph-VySiMe_3_.

**Figure S4.** GPC for Me_3_SiVy-Terph-VySiMe_3_.

**Figure S5.** GPC for Me_3_SiVy-Stil-VySiMe_3_.

**Figure S6**. GPC for Me_3_SiVy-Me_2_Fl-VySiMe_3_.

**Figure S7**. GPC for Me_3_SiVy-Thio-VySiMe_3_.

**Figure S8**. GPC for Me_3_SiVy-Bithio-VySiMe_3_.

**Figure S9**. GPC for Me_3_SiVy-Ththio-VySiMe_3_.

**Figure S10.** GPC for the Vy_4_D_4_-coPh.

**Figure S11.** GPC for the Vy_4_D_4_-coBiph.

**Figure S12.** GPC for the Vy_4_D_4_-coTerPh.

**Figure S13.** GPC for the Vy_4_D_4_-coMe_2_Fl.

**Figure S14.** GPC for the Vy_4_D_4_-coStil.

**Figure S15**. GPC for the Vy_4_D_4_-coThio.

**Figure S16.** GPC for the Vy_4_D_4_-coBithio.

**Figure S17.** GPC for the Vy_4_D_4_-coThthio.

**Figure S18.** GPC for the (Vy_4_D_4_-Thio)-coPh.

**Figure S19.** GPC for the (Vy_4_D_4_ -Thio)-coBiph.

**Figure S20.** GPC for the (Vy_4_D_4_-Thio)-coTerph.

**Figure S21**. GPC for the (Vy_4_D_4_-Thio)-coMe_2_Fl.

**Figure S22**. GPC for the (Vy_4_D_4_-Bithio)-coPh.

**Figure S23**. GPC for the (Vy_4_D_4_-Bithio)-coBiph.

**Figure S24**. GPC for the (Vy_4_D_4_-Bithio)-coTerph.

**Figure S25**. GPC for the (Vy_4_D_4_-Bithio)-coMe_2_Fl.

**Figure S26.** GPC for the (Vy_4_D_4_-Terph)-coThio.

**Figure S27.** GPC for the (Vy_4_D_4_-Terph)-coBithio.

**Figure S28.** GPC for the (Vy_4_D_4_-Stil)-coThio.

**Figure S29.** GPC for the (Vy_4_D_4_-Stil)-coBithio.

**Figure S30.** GPC for the (Vy_4_D_4_-Me_2_Fl)-coThio.

**Figure S31.** GPC for the (Vy_4_D_4_-Me_2_Fl)-coBithio.

**Figure S32.** GPC for the (Vy_4_D_4_-Me_2_Fl)-coThthio.

**Figure S33.** GPC blank for THF. The peak at 10.9 min is attributed to the stabilizer butylated hydroxytoluene (BHT) present in the THF solvent.

**FTIR**

**Figure S34**. FTIR for silane model compounds (Ph, BiPh, Terph, Stil, Me_2_Fl).

**Figure S35**. FTIR for silane model compounds (Thio, Bithio, Ththio).

**Figure S36**. FTIR for Vy_4_D_4_-copolymers (Ph, BiPh, Terph, Stil, Me_2_Fl).

**Figure S37**. FTIR for Vy_4_D_4_-copolymers (Thio, Bithio, Ththio).

**Figure S38**. FTIRs of decorated copolymers (with thiophene pendants).

**Figure S39**. FTIR of decorated copolymers (with bithiophene pendants).

**Figure S40**. FTIR for decorated copolymers (with terphenyl pendants).

**Figure S41**. FTIR for decorated copolymers (with stilbene pendants).

**Figure S42**. FTIR for decorated copolymers (with dimethylfluorene pendants).

**NMRs**

**Figure S43**. ^1^H NMR for Vy_4_D_4_-coPh.

**Figure S44**. ^1^H NMR for Vy_4_D_4_-coBiph.

**Figure S45**. ^1^H NMR for Vy_4_D_4_-coTerph.

**Figure S46**. ^1^H NMR for Vy_4_D_4_-coStil.

**Figure S47**. ^1^H NMR for Vy_4_D_4_-coMe_2_Fl.

**Figure S48**. ^1^H NMR for Vy_4_D_4_-coThio.

**Figure S49**. ^1^H NMR for Vy_4_D_4_-coBithio.

**Figure S50**. ^1^H NMR for Vy_4_D_4_-coThthio.

**Figure S51**. ^13^C NMR for Vy_4_D_4_-coPh.

**Figure S52**. ^13^C NMR for Vy_4_D_4_-coBiph.

**Figure S53**. ^13^C NMR for Vy_4_D_4_-coTerph.

**Figure S54**. ^13^C NMR for Vy_4_D_4_-coStil.

**Figure S55**. ^13^C NMR for Vy_4_D_4_-coThio.

**Figure S56**. ^13^C NMR for Vy_4_D_4_-coBithio.

**Figure S57**. ^13^C NMR for Vy_4_D_4_-coThthio.

**TGA-DSC**

**Figure S58.** TGA for Vy_4_D_4_-coPh.

**Figure S59.** TGA for Vy_4_D_4_-coBiph.

**Figure S60.** TGA for Vy_4_D_4_-coTerph.

**Figure S61.** TGA for Vy_4_D_4_-coStil.

**Figure S62.** TGA for Vy_4_D_4_-coMe_2_Fl.

**Figure S63.** TGA for Vy_4_D_4_-coThio.

**Figure S64.** TGA for Vy_4_D_4_-coBithio.

**Figure S65.** TGA for Vy_4_D_4_-coThthio.

**Figure S66.** TGA for (Vy_4_D_4_-Thio)-coPh.

**Figure S67.** TGA for (Vy_4_D_4_-Thio)-coBiph.

**Figure S68.** TGA for (Vy_4_D_4_-Thio)-coTerph.

**Figure S69.** TGA for (Vy_4_D_4_ -Thio)-coMe_2_Fl.

**Figure S70.** TGA for (Vy_4_D_4_-Bithio)-coPh.

**Figure S71.** TGA for (Vy_4_D_4_ -Bithio)-coBiph.

**Figure S72.** TGA for (Vy_4_D_4_ -Bithio)-coTerph.

**Figure S73.** TGA for (Vy_4_D_4_-Bithio)-coMe_2_Fl.

**Figure S74.** TGA for (Vy_4_D_4_-Terph)-coThio.

**Figure S75.** TGA for (Vy_4_D_4_-Terph)-coBithio.

**Figure S76.** TGA for (Vy_4_D_4_-Stil)-coThio.

**Figure S77.** TGA for (Vy_4_D_4_-Stil)-coBithio.

**Figure S78.** TGA for (Vy_4_D_4_ -Me_2_Fl)-coThio.

**Figure S79.** TGA for (Vy_4_D_4_-Me_2_Fl)-coBithio.

**Figure S80.** TGA for (Vy_4_D_4_-Me_2_Fl)-coThthio.

Photophysical properties

**Figure S81**. Normalized absorption (in solid lines) and emission (in dashed lines) for trimethyl silane model compounds (in red) vs the corresponding methoxyl silane model compounds (in blue). (a) Ar = benzene, (b) Ar=biphenyl, (c) Ar=terphenyl, (d) Ar=stilbene, (e) Ar=dimethylfluorene, (f) Ar=thiophene, (g) Ar=bithiophene, (h) Ar=thienothiophene.

**Figure S82**. Two-dimensional excitation-emission map for Vy_4_D_4_-co-benzene in acetonitrile (upper panel) and Vy_4_D_4_-co- dimethylfluorene in DCM (lower panel). The same data is plotted on linear (left) and on logarithmic intensity scale (right). The two origins are observed at 325 and 375 nm for Vy_4_D_4_-co-benzene and at 370 and 430 nm for Vy_4_D_4_-co- dimethylfluorene. Diagonal lines correspond to the 1^st^ and 2^nd^ order excitation wavelength scattering artifacts.

**Figure S83**. Time-resolved emission for Vy_4_D_4_-co-benzene in acetonitrile (upper panel) and Vy_4_D_4_-co-dimethylfluorene in DCM (lower panel) measured using a short wavelength pass (upper graphs) and long wavelength pass (lower graph) emission spectral filters. Graphs on the left show the corresponding filtered spectra (tan) along with non-filtered emission spectrum (blue), where the first roughly match the corresponding shorter- and longer-wavelength emission components of the two compounds.
